# Tumour heterogeneity and personalized treatment screening based on single-cell transcriptomics

**DOI:** 10.1016/j.csbj.2024.12.020

**Published:** 2024-12-25

**Authors:** Xinying Zhang, Jiajie Xie, Zixin Yang, Carisa Kwok Wai Yu, Yaohua Hu, Jing Qin

**Affiliations:** a School of Pharmaceutical Sciences (Shenzhen), Shenzhen Campus of Sun Yat-sen University, Shenzhen, Guangdong 518107, China; b Department of Mathematics, Statistics and Insurance, The Hang Seng University of Hong Kong, Shatin, Hong Kong; c School of Mathematical Sciences, Shenzhen University, Shenzhen, Guangdong 518060, China

**Keywords:** Tumour heterogeneity, Single-cell transcriptomes, Individualized therapy, Tumour biomarker, Drug repurposing

## Abstract

According to global cancer statistics for the year 2022, based on updated estimates from the International Agency for Research on Cancer, there were approximately 20 million new cases of cancer in 2022 alongside 9.7 million related deaths. Lung, breast, colorectal, gastric, and liver cancers are the most common types of cancer. Despite advancements in anticancer drugs and optimised chemotherapy regimens that have improved cure rates for malignant tumours, the presence of tumour heterogeneity has resulted in substantial variations among patients in terms of disease progression, clinical response, sensitivity to therapy, and prognosis, posing significant challenges in attaining optimal therapeutic outcomes for each patient. Here, we collected five single-cell transcriptome datasets from patients with lung, breast, colorectal, gastric, and liver cancers and constructed multiple cancer blueprints of tumour cell heterogeneity. By integrating multiple bioinformatics analyses, we explored the biological differences underlying tumour cell heterogeneity at the single-cell level and identified tumour cell subcluster-specific biomarkers and potential therapeutic drugs for each subcluster. Interestingly, although tumour cell subpopulations exhibit dramatic differences within the same cancer type and between different cancers at both the genomic and transcriptomic levels, some demonstrate similar oncogenic pathway activities and phenotypes. Tumour cell subpopulations from the five cancers listed above were classified into three major groups corresponding to different treatment strategies. The findings of this study not only focus on the differences but also on the similarities among tumour cell subpopulations across different cancers, providing new insights for individualised therapy.

## Graphical Abstract

**Figure:**
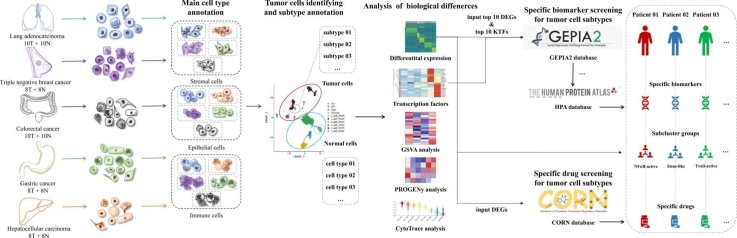

## 1 Introduction

Cancer is a leading cause of death worldwide. According to global cancer statistics for the year 2022, based on updated estimates from the International Agency for Research on Cancer (IARC), there were approximately 20 million new cases of cancer in 2022 and 9.7 million deaths associated with it [1]. Although the development of anticancer drugs and optimisation of chemotherapy regimens have led to improved treatment outcomes for malignant tumours, significant differences exist among patients in terms of disease progression, clinical response, sensitivity to radiation and chemotherapy, and prognosis due to the presence of tumour heterogeneity, posing a challenge in achieving optimal therapeutic outcomes for each patient. Therefore, there is an urgent need to develop more effective anticancer drugs and therapies. However, in the traditional clinical and histopathological classification of cancer, guidance provided by morphological features based on imaging and microscopic cell morphology is currently limited. It fails to accurately capture and predict the clinical manifestations of each tumour at the cellular and molecular levels, making it difficult to select personalised treatment plans. As a result, there is a growing need to explore alternative approaches to identify and classify tumours to gain a better understanding of their underlying pathological mechanisms and provide accurate personalised treatment strategies.

Tumour heterogeneity is typically categorised into two types: 1) intratumoral heterogeneity, which refers to differences among diverse tumour subpopulations across different or within a single disease site in one patient; and 2) intertumoral heterogeneity, which refers to that among patients with the same histological type of tumour [2]. Intratumoral heterogeneity can be defined as temporal or spatial. Temporal heterogeneity refers to the presence of tumours that emerge at different stages of the disease progression and is attributed to dynamic variations in the genetic diversity of an individual tumour over time [2]. For example, compared to early stage, late-stage tumours may exhibit stronger selection for survival strategies, such as enhanced invasion and angiogenesis, due to nutrient depletion and hypoxic conditions [3]. Spatial heterogeneity, on the other hand, refers to the uneven distribution of genetically diverse tumour cells within a single disease site or between different ones [4]. Inter-tumoural heterogeneity is believed to arise from a combination of patient-specific factors, including germline genetic variations, differences in somatic mutation profiles, and environmental factors [2]. Inter-tumoural heterogeneity is a critical barrier to current precision medicine practices. For example, researchers have found that lung adenocarcinoma (LUAD) exhibits genetic mutation heterogeneity and significantly altered pathways across different subtypes [5]. Specifically, the invasive adenocarcinoma subtype shows a significant enrichment of mutations in genes related to the mTOR and Hippo signalling pathways. Different mutations contribute to the potential mechanisms underlying different prognoses in different LUAD cohorts, emphasising the necessity for individualised clinical management of different subtypes. Therefore, by exploring heterogeneity, we can gain a better understanding of tumour diversity and provide patients with more effective treatment strategies, ultimately improving treatment outcomes and survival rates.

High-throughput sequencing technologies such as bulk transcriptome sequencing have been employed to investigate tumour heterogeneity. Via bulk RNA sequencing (RNA-seq) of tumour tissues, researchers can obtain an overall characterisation of millions of cells in a tumour sample. This contributes to the screening of tumour-associated mutated genes and signalling pathways [6]. Through transcriptome and genome analyses, various cancers can be classified into several molecular subtypes based on their consensus genes. Each molecular subtype has different biological characteristics and prognoses, with varying responses to treatment, significantly driving the advancement of personalised medicine in clinical practice [7], [8], [9]. However, bulk RNA-seq typically detects average signals from mixed populations of cells rather than signals from individual cells within the tissue and is unable to differentiate the proportions of different cellular components within the tumour [10]. In contrast, single-cell RNA sequencing (scRNA-seq) enables the sequencing of gene expression information from individual cells at unprecedented resolution. It can capture the transcriptional differences between different cells and preserve heterogeneity within the tumour. Recently, scRNA-seq has been successfully used in many cancers and has shown tremendous advantages for analysing the molecular and biological characteristics of tumour cells. Dai *et al.* performed scRNA-seq analysis of 2824 cells from patients with stage Ⅲ colorectal cancer (CRC). They clustered cells into five distinct clusters and found that the highly expressed genes in each cluster were enriched in different ontological pathways. In addition, some highly expressed genes identified through scRNA-seq were not detected in the bulk transcriptome analysis, suggesting that scRNA-seq provides a more comprehensive assessment of potential pathological processes at the molecular level in different patients or tumour sites [11]. The success of cancer immunotherapy has inspired an in-depth exploration of the holistic tumour ecosystem, particularly the immune aspects of the tumour microenvironment. Using scRNA-seq technology, different subsets of each component in the tumour microenvironment have been identified, allowing for a more detailed understanding of their interactions and functions in cancer development [12], [13], [14], [15]. Remarkably, a team led by Prof. Zemin Zhang constructed single-cell atlases for various immune cells, including T [16], B [17], myeloid [18] and natural killer [19] cells, revealing the intricate interactions and heterogeneity within the tumour microenvironment and mechanisms of tumour immune evasion. In summary, the significant advantages of scRNA-seq technology in studying tumour heterogeneity have made it an indispensable tool for investigating the tumour microenvironment and mechanisms of cancer development. However, as mentioned above, the majority of recent studies are primarily centred on the tumour microenvironment to dissect its cellular diversity and complexity, with limited research specifically targeting the epithelial cells of epithelial-derived cancers. Tumour cells from various epithelial-derived cancers exhibit a high degree of specificity, and studying their heterogeneity can aid in early tumour diagnosis, prevention, and development of more effective personalised treatment strategies to improve therapeutic outcomes and survival rates. In addition, single-cell studies have revealed diverse patterns of heterogeneity in various cancers, extending beyond immune cells, and have classified cancers into distinct subtypes. These findings are useful for understanding tumour heterogeneity. Zhang *et al.* classified malignant gastric cells into five subgroups (C1-C5) based on transcriptomic characteristics and observed variations in the degree of differentiation among these subgroups. The differentiation degree was correlated with patient outcomes, thereby enhancing the precision of gastric cancer diagnosis and prognosis evaluation in clinical practice [20]. Similarly, Guo *et al.* identified three subtypes of hepatic carcinoma cells: the ARG1 metabolism subtype (Metab subtype), TOP2A proliferation phenotype (Prol phenotype), and S100A6 pro-metastatic subtype (epithelial-to-mesenchymal transition (EMT) subtype). Enrichment analysis revealed that the three subtypes harboured different features: metabolism, proliferation, and EMT, respectively [21]. The criteria for classifying and identifying heterogeneous tumour subtypes vary across studies, with many focusing solely on a single type of cancer. Previous researchers have employed scRNA-seq to analyse 198 cancer cell lines across 22 cancer types, identifying 12 recurrent expression programs associated with multiple biological processes within numerous cell lines and elucidating the common patterns of cellular heterogeneity in pan-cancer [22]. Presently, translating these similar biological phenotypic patterns into appropriate clinical treatment plans remains challenging.

This study aimed to transcend the traditional one-size-fits-all approach to cancer treatment. By harnessing the power of personalised medicine, we sought to craft bespoke strategies for each patient, considering the unique characteristics of their tumours. However, the development of unique drugs for each patient is not time- and cost-effective, or practical. To address these challenges, we explored the intricate heterogeneity among tumour cells across various types of cancer to elucidate the inter and intratumoral heterogeneity referring to the heterogeneity among patients with a single type of cancer, and that within the same patient, respectively, to uncover the underlying biological differences and explore corresponding potential treatments. Our research examined single-cell transcriptome datasets sourced from public databases encompassing a spectrum of malignancies, including lung, breast, colorectal, gastric, and liver cancers. We uncovered some interesting patterns across cancer types and classified tumour cell subclusters from five cancers into three major groups corresponding to different treatment strategies, proposing a new perspective for classifying cancer patients and developing targeted treatment plans.

## 2 Material and methods

### 2.1 Data collection and experiment design

**Fig. 1:**
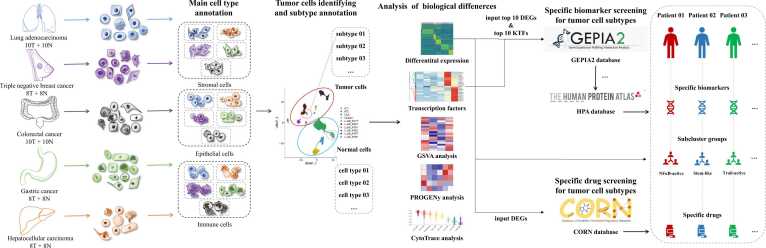
Analysis workflow of tumour and matched normal samples from five cancer types. The single-cell transcriptome sequencing data of five different cancers were collected from public databases, GEO and OMIX. These single-cell RNA sequencing datasets were analysed using the same set of pipelines. In brief, after the quality control, filtering, normalization, batch effect correction, and dimensionality reduction, single cells of each cancer type from different sample sources were integrated and clustered into three main cell types. Epithelial cells were further classified with higher solutions to identified tumour cell subclusters. Biological differences among tumour cell subclusters were investigated in terms of copy number variation, gene expression, transcription regulation, signalling pathway, and stemness. Then subcluster-specific biomarkers and drugs were screened using various databases. [Abbreviations: T, tumour samples; N, normal samples; DEG, differentially expressed gene; KTF, key transcription factor].

Single-cell transcriptome sequencing data of five cancers were collected from the public databases Gene Expression Omnibus [23] (GEO, https://www.ncbi.nlm.nih.gov/geo/) and the Open Archive for Miscellaneous Data (OMIX, https://ngdc.cncb.ac.cn/omix/). The detailed data are shown in Table S1. These scRNA-seq datasets were analysed using the same set of pipelines, as shown in Fig. 1. Briefly, after quality control, filtering, normalisation, batch effect correction, and dimensionality reduction, single cells of each cancer type from different sample sources were integrated and clustered into three main cell types. Epithelial cells were further classified into higher solutions to identify tumour cell subclusters. Biological differences among tumour cell subclusters were investigated in terms of copy number variation (CNV), gene expression, transcriptional regulation, signalling pathways, and stemness. Sub-cluster-specific biomarkers and drugs were screened using various databases.

### 2.2 Filtering and normalization

The R package Seurat [24] was used to remove low-quality cells and genes using the following filtering criteria: cells with 500–9000 genes, 1000–100,000 UMI, fraction of mitochondrial genes < 20 %, and log10GenesPerUMI > 0.8. The filtered genetic barcode matrix was standardised using the normalised data function in Seurat with default parameters to eliminate the influence of varying sequencing depths across different cells. Next, the FindVariableFeatures function was used to identify the top 2000 most variable genes for principal component analysis (PCA) dimension reduction. Because cell cycle-related genes can affect the effectiveness of dimension reduction and clustering, the CellCycleScoring function was used to calculate the cell cycle scores, and the ScaleData function was employed to normalise the data. The normalised gene expression values were transformed into Z-scores, ensuring that the mean expression of each gene across all cells was 0 and the variance was 1. The vars.to.regress parameter was set to correct for the effects of mitochondrial genes, cell cycle, and other sources of variation on subsequent analyses.

### 2.3 Dimension reduction

To determine the number of principal components (PCs), we first calculated the variance and cumulative variance percentages for each PC. We selected the first PC where the cumulative variance percentage exceeded 90 % and ensured that the difference in the variance percentage between this and the next PC was less than 5 %. Additionally, we considered the last PC when the difference in the percentage of variance between adjacent PCs was greater than 0.1 %. Ultimately, we used the minimum number of PCs determined by these two criteria to ensure that the main variations in the data were retained while avoiding overfitting.

### 2.4 Clustering and cell type annotation

The R package Harmony [25] was used for data integration to eliminate batch effects between patients and sequencing batches. The FindNeighbors and FindClusters functions in the Seurat package were then applied to perform graph-based clustering on the data after dimension reduction using PCA. Nonlinear dimension reduction was applied using the RunUMAP function, and the results for the two-dimensional Uniform Manifold Approximation and Projection were visualised using the Dimplot function. A resolution of 0.5 was applied to cluster the main cell types and a resolution of 2 was applied for the subcluster annotation of epithelial cells. The cell type annotations were based on the composition of the tumour microenvironment, and the cells were classified into three major cell types based on classical marker genes: epithelial (EPCAM, SFN, and KRT19), immune (PTPRC, CD3E, and CD79A), and stromal cells (PECAM1, CD34, VWF, ACTA2, FAP, and THY1) [26], [27], [28], [29]. For the epithelial cell subtype annotation, dimensional reduction and reclustering were performed. Subsequently, the epithelial cells were re-annotated based on marker genes specific to epithelial cell subtypes in the lung, breast, colorectal, gastric, and liver from the existing literature. Marker genes for different epithelial cell types in various organs are shown in Table S2.

### 2.5 Copy number variation analysis

The CNV status of each epithelial cell was estimated using the InferCNV R package, which can be used to distinguish normal cells from tumour cells because tumour cells tend to have more CNVs [30]. The CNV score of each cell was calculated as described by Peng *et al.* [31]. Briefly, after subtracting 1 from the scores obtained by the InferCNV package, the sum of squares was calculated and divided by the number of genes. Clusters with CNV scores higher than the average were considered tumour cell clusters, whereas those below were considered normal cell clusters.

### 2.6 Differential gene expression analysis

Differentially expressed genes (DEGs) between patient-specific tumour epithelial cells and corresponding normal epithelial cells were computed using the FindMarkers function with the following parameters: fraction of marker expressing cells ≥ 0.1, log2 fold change between cell populations ≥ 0.25, adjusted p-value < 0.05, and Model-based Analysis of Single-cell Transcriptomics method was selected. This is a statistical method specifically designed to analyse scRNA-seq data [32]. It employs a hierarchical model that accounts for the unique characteristics of single-cell expression data, such as zero inflation. Visual heat maps of the top ten sub-cluster-specific DEGs were generated using the DoHeatmap function. The identified DEGs and their log2 fold change values were input into the matching tool of Condition Orientated Regulatory Networks (CORN) [33].

### 2.7 Transcription factor activity analysis

Transcription factor (TF) activity was analysed using SCENIC [34] and pySCENIC [35] per subtype, with expression raw count matrices as input. Differentially activated TFs of each subtype were identified using the Wilcoxon rank-sum test. We identified specific key TFs for different cellular sub-clusters and their corresponding target genes within the regulons using pySCENIC. Subsequently, we intersected these target genes with the Cancer Gene Census from the Catalogue of Somatic Mutations in Cancer (COSMIC) database [36], aiming to illustrate the cancer genes regulated by subcluster-specific key TFs. The top 10 key TFs of each subtype were considered potential biomarkers for subsequent analysis.

### 2.8 Gene set variation analysis

Enrichment analyses were conducted using the gene set variation analysis (GSVA) R package [37] with the hallmark gene set. This quantifies the activity of functional pathways in different tumour cell subtypes. Heatmaps were generated to visualise the activity of each of the 50 pathways derived from the hallmark gene sets, which encapsulated distinct, well-characterised biological conditions or processes and exhibited consistent patterns of gene expression.

### 2.9 Oncogenic signalling pathway activity analysis

In order to further assess the differences in oncogenic signalling pathways among different tumour cell subtypes, we utilised the R package PROGENy [38] to evaluate the activity of cell subtypes on 14 classical oncogenic pathways (Androgen, Oestrogen, Hypoxia, EGFR, STAT, MAPK, NFκB, PI3K, p53, TNF-α, TGF-β, Trail, VEGF, and Wnt). The sample data were first subjected to Z-score normalisation prior to the analysis. Heatmaps were generated to visualise the activity of oncogenic pathways across the different tumour subtypes.

### 2.10 Cellular differentiation status analysis

The R package CytoTRACE [39] was applied to predict differentiation scores. CytoTRACE is a computational tool designed to analyse cellular trajectories and developmental processes using single-cell RNA-sequencing data. This tool allows researchers to elucidate the underlying dynamics of cell differentiation and lineage progression by quantifying the developmental trajectories of individual cells. A higher score indicated less differentiation and greater stemness.

### 2.11 Biomarker screening

The Gene Expression Profiling Interactive Analysis (GEPIA2) database [40] (http://gepia2.cancer-pku.cn/) is a valuable online tool that facilitates gene expression analysis using data from The Cancer Genome Atlas (TCGA) and Genotype-Tissue Expression (GTEx) projects. The GEPIA2 database was used to investigate the correlation between the expression of specific genes (DEGs and key TFs identified above) and patient prognosis. The Human Protein Atlas (HPA) database [41] (https://www.proteinatlas.org) is a comprehensive resource that provides information on the expression and localisation of proteins in human tissues and organs. It combines data from various omics technologies including immunohistochemistry, mass spectrometry, and transcriptomics to create a detailed map of the human proteome. The HPA database was used to explore the protein expression patterns of highly expressed genes and key TFs in tumour and normal tissues. Genes or TFs with survival analysis results consistent with their protein expression levels were considered biomarkers for tumour cell subclusters.

To substantiate the validity and utility of the biomarkers we delineated, we performed external validation on an independent dataset devoid of TCGA and GTEx project data, sourced from GEPIA2. To this end, we employed the Kaplan-Meier plotter [42], [43], [44] (https://kmplot.com/analysis/) to delineate survival curves for biomarkers specific to lung adenocarcinoma (LUAD), breast (BC), colorectal (CRC), and gastric (GC) cancers. Notably, given the presence of TCGA data within the hepatocellular carcinoma (HCC) dataset in the KM plotter database, we opted to utilise the PanCanSurvPlot database [45] (https://smuonco.shinyapps.io/PanCanSurvPlot/) to validate HCC-specific biomarkers. This approach ensured the robustness of our findings by corroborating them against a distinct set of data, thereby enhancing the generalisability of our biomarker discovery.

### 2.12 Drug screening

CORN (https://qinlab.sysu.edu.cn/corn/home) is a library of condition-based (natural compound/small molecule/drug treatments and gene perturbations) transcriptional regulatory subnetworks (TRSNs) that come with an online TRSN matching tool [33]. CORN associates 7540 specific conditions with 71934 TRSNs in 52 human cell lines, involving 542 TFs. The online TRSN matching tool on the CORN website allows users to identify the closest complementary TRSNs with a set of input DEGs and the change in their expression in a disease. Thus, the associated condition can be considered a candidate to regulate or even cure the abnormal cell state. To identify the TRSN that best matches the inputted transcriptomic changes and pinpoint the specific condition corresponding to it, a sparse learning model adaptive FoBa (Forward-Backward) greedy algorithm was employed under the assumption that ‘disturbances in the transcriptomes of a disease could be reversed by a few condition-specific TRSNs’. The matching score is used to reflect the contributions of different TRSNs to input transcriptome changes. In short, the larger the magnitude of the resulting matching score, the closer the resemblance between TRSN and the differential expression profile. Focusing on TRSNs with positive scores would help identify conditions/drugs that can potentially reverse or mitigate the effects of disease-related gene expression alterations.

## 3 Results

### 3.1 The exploration of tumour cell heterogeneity

Single-cell transcriptome sequencing data for five cancers were collected from the public databases, GEO and OMIX, covering LUAD, BC, CRC, GC, and hepatocellular carcinoma (HCC), including cancer tissue samples and their corresponding adjacent normal tissue samples (Table S1). After quality control, batch effect correction, and dimension reduction, the single-cell transcriptome data for each cancer type from the different sample sources were well integrated (Fig. S1). For each cancer type, over 30,000 cells from both normal and tumour tissue samples from multiple patients were grouped into clusters (Fig. S2). These cells are categorised into three primary types: stromal, epithelial, and immune cells (Fig. S3). The detailed process is illustrated in Fig. 1.

**Fig. 2:**
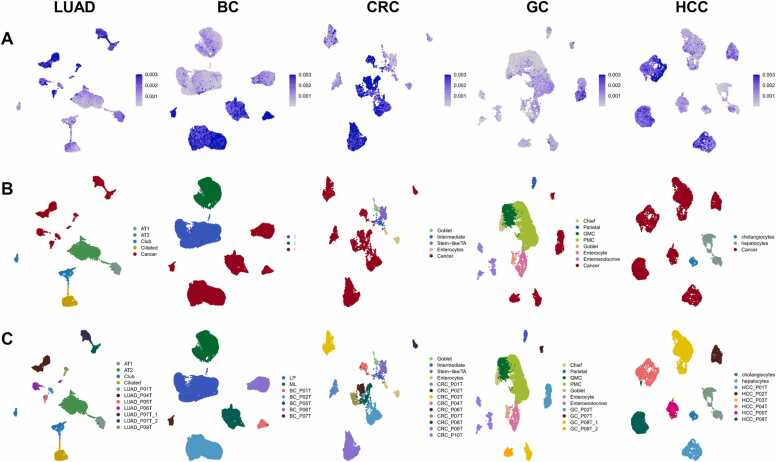
Uniform manifold approximation and projection based on the epithelial cells of all single-cell transcriptomes from five cancer types, color-coded by (A) copy number variation score, (B) cell type and (C) subtype. [Abbreviations: AT1, type Ⅰ alveolar epithelial cells; AT2, type Ⅱ alveolar epithelial cells; LP: luminal progenitor cells; ML: mature luminal cells; Stem-like/TA: stem-like/transit amplifying; GMC: gland mucous cells; PMC: pit mucous cells; LUAD_P01T, tumour cells of lung adenocarcinoma patient 01].

Because the majority of tumour cells in these five cancers originate from the malignant transformation of normal epithelial cells, we specifically focused on analysing epithelial cell populations to further investigate the heterogeneity of tumour cells. Therefore, epithelial cells from the single-cell transcriptome atlas were extracted and clustered again into epithelial cell subtypes with higher resolution. Based on the CNV score of each cell (Fig. 2A) and the marker genes for epithelial cell subtypes listed in Table S2, various normal epithelial cell types and tumour cell subpopulations from the five organs were identified (Fig. 2B). Interestingly, when the single-cell epithelial maps were colour-coded per patient, normal epithelial cells from different patients were intermingled (Fig. 2B-C). This observation suggests that batch-effect correction effectively removed genetic background differences and technical errors among patients. In contrast, the tumour cells displayed a significant level of patient-specific clustering, underscoring the existence of substantial intertumoural heterogeneity (Fig. 2C). Moreover, intratumoral heterogeneity was evident in certain cases, such as in LUAD_P07 and GC_P08, where tumour cells were divided into two distinct cell clusters (LUAD_P07T_1, LUAD_P07T_2, GC_P08T_1, and GC_P08T_2) (Fig. 2C). For patient 08 with GC, two spatial sites were collected because of the large tumour size. In addition, most of our samples were obtained using single-region sampling. There was limited heterogeneity among the tumour cells within the adjacent area, resulting in fewer cases of intratumour heterogeneity in the collected data. To better demonstrate the biological differences between these patient-specific tumour cell subpopulations, we named the tumour cell subclusters according to their origins (Fig. 2C).

### 3.2 Biological differences among tumour cell subclusters

**Fig. 3:**
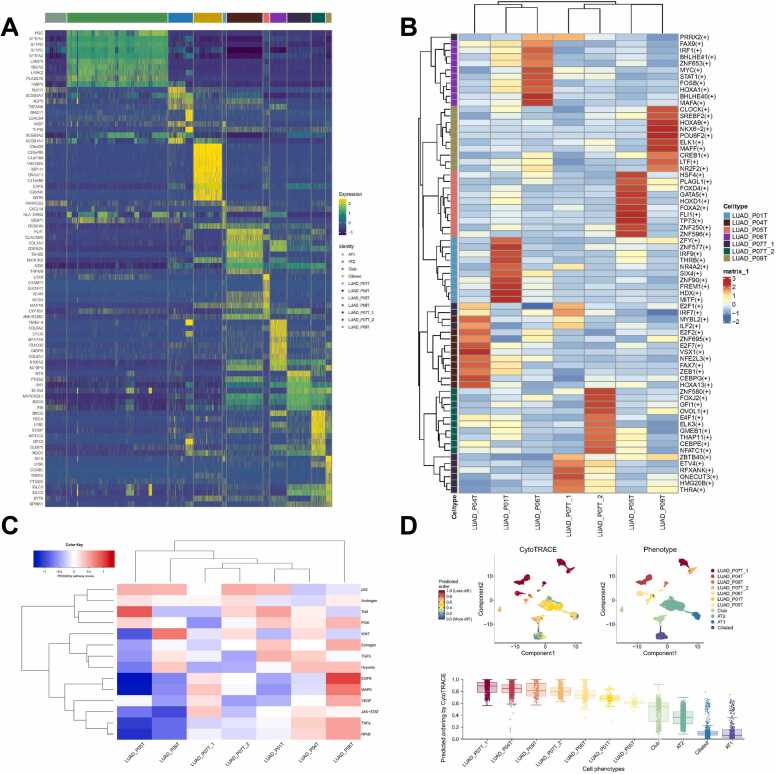
Biological differences among tumour cell subtypes from lung adenocarcinoma. Heatmaps of (A) the top 10 differentially expressed genes, (B) the top 10 key transcription factors, and (C) PROGENy pathway activity in each tumour cell subtype. (D) Uniform manifold approximation and projection and box chart based on differentiation potential score. See supplementary materials for the other four cancer types.

In this section, we investigate the biological differences among tumour cell subclusters across the five cancer types. Interestingly, although the genomic and transcriptomic variations among different patients exhibited heterogeneity, we discovered similar patterns in the activated oncogenic pathways, even among different cancer types. These findings provide a basis for the classification of clinical treatments. To investigate the biological differences among tumour cell sub-clusters, we started from their genomic variations, in which genomic CNVs play a crucial role in tumour cell evolution. As key factors in tumour cell heterogeneity, CNVs inferred from scRNA-seq exhibited substantial differences among tumour cell subpopulations with the same type of cancer (Figs. S9-S13). Genomic variations can lead to transcriptome changes in different ways; for example, copy number changes in genes located in CNV regions can directly change their transcription levels or cause mutations within TFs and their binding sites, as well as TF expression variations, which may affect the expression of their target genes. This prompted us to identify patient-specific DEGs between normal epithelial cells and tumour subpopulations, which revealed unique expression profiles for each subcluster (Fig. 3A, Figs. S14-S17A). Further analysis using the SCENIC software package highlighted the importance of TFs in mediating changes in gene expression (Fig. 3B, Figs. S14-S17B, Table S5), and cancer genes regulated by specific key TFs (Table S6). We used the PROGENy package and GSVA to assess the heterogeneity of signalling pathways across various cancer cell subclusters (Fig. 3C and Figs. S14-S17C, S18-S22). Furthermore, we used CytoTRACE to estimate stemness across individual cells, because cancer stemness is reported to be a critical factor in tumour development. Our results showed that the majority of tumour cell subclusters had a higher stemness than normal epithelial cell types, while some of them had a higher stemness than others (Fig. 3D and Figs. S14-S17D). These findings emphasise the complexity of tumour cell heterogeneity and suggest potential therapeutic targets that consider this diversity.

#### 3.2.1 Lung adenocarcinoma

InferCNV analysis revealed significant chromosomal amplification in tumour cells from different patients with LUAD. Specifically, the cells from patient LUAD_P05T showed substantial amplification of chromosome 1. In contrast, LUAD_P04T and LUAD_P07T exhibited notable amplification on chromosomes 7 and 17, respectively (Fig. S9 for the visual representations). DEGs between normal epithelial cells and each tumour cell subpopulation were identified, some of which may have originated from CNVs in the genome. For example, FSCN1, a gene located on chromosome 7, was found to have an increased copy number and differential expression in LUAD_P04. Similarly, MFSD4, a DEG in LUAD_P05 located within the CNV region of chromosome 1, also showed an increased copy number. Key TFs regulating the DEGs were also searched using SCENIC for each tumour subpopulation. Heatmaps show the top 10 specifically highly expressed genes along with the top 10 key TFs, some of which have been confirmed to be associated with cancer (Fig. 3A-B). The LUAD_04T subcluster has a high differential expression of CEACAM5 (also known as CEA), a member of the carcinoembryonic antigen-related cell adhesion molecule (CEACAMs) family, which has already been identified and used as a tumour biomarker [46], [47]. The LUAD_P06T sub-cluster specifically overexpresses EREG and SPRR1B. Researchers have previously found that these two genes can activate the EGFR and MAPK pathways, respectively, which promote tumour cell proliferation and development and may lead to a poorer prognosis [48], [49]. This is consistent with the results of the PROGENy analysis (Fig. 3C), which demonstrated that the EGFR and MAPK pathways were the two most activated oncogenic signalling pathways in LUAD_P06T cells.

#### 3.2.2 Breast cancer

Fig. S14A presents a heatmap depicting the top 10 DEGs across various breast cancer tumour cell subclusters, whereas Fig. S14B displays the SCENIC analysis results, highlighting the unique key TFs for these subclusters. These visualisations revealed distinct patterns of gene expression and regulation. Notably, certain cancer-related genes and TFs exhibited significant differential expression. For example, the tumour cell subpopulation BC_P02T demonstrated a high level of KRT14 [50], while the BC_P06T subpopulation was marked by pronounced expression of FABP7 [51], highlighting heterogeneity. Similarly, the BC_P05T subcluster is characterised by elevated KRT5 expression and is primarily regulated by the transcription factor HOXB2, which restricts the occurrence of triple-negative breast cancer by reshaping the extracellular matrix [52], [53]. Variations in the expression of some DEGs from various BC tumour cell subclusters may originate from CNVs in the genome. For example, BC_P01 showed specific abnormal CNVs on chromosomes 3, 6, 8, and 22 (Fig. S10). HSPA1B is a highly expressed DEG in the BC_P01T subcluster located in the CNV region of chromosome 6. BC_P05 exhibited abnormal CNVs on chromosomes 2, 3, 19, and 21. TSEN34 is a highly expressed DEG in the BC_05T subcluster located in the CNV region of chromosome 19. Both genes exhibited high CNV scores. Then GSVA and PROGENy results revealed significant variations in the activity levels of the JAK/STAT, p53, NFκB, and TNF-α signalling pathways among distinct tumour cell subclusters (Fig. S14C, Fig.S19). CytoTRACE analysis showed that among normal epithelial cells, luminal progenitor cells had a higher differentiation potential than mature luminal cells, as expected. Notably, the tumour cell subpopulations exhibited even higher scores, indicating higher stemness than that of normal epithelial cells (Fig.S14D). This aligns with the GSVA results (Fig. S19). Various tumour cell subpopulations were enriched in cell cycle-related signalling pathways, such as the G2/M checkpoint, MYC targets, and p53, as well as in EMT processes and the hedgehog pathway, which are known to play crucial roles in regulating stemness and cellular differentiation.

#### 3.2.3 Colorectal cancer

CRC cells from different patients also showed heterogeneity at both the genomic and transcriptomic levels. Fig. S15A, B, Table S3, and Table S5 illustrate the DEGs and key TFs of various tumour cell subclusters in CRC. Both CRC_02 and CRC_P09 exhibited significantly abnormal gene amplifications on chromosome 20 (Fig. S11). This prompted us to examine the DEGs of tumour cell subclusters from these two patients. We found that SDC4, a DEG of the subcluster CRC_P09T, and TNNC and FERMT1, DEGs of the subcluster CRC_P02T, were all located on chromosome 20 and had high CNV scores. Among the tumour cell subclusters, CRC_P01T, CRC_P03T, and CRC_P07T exhibited notably higher Wnt signalling pathway scores in the PROGRNy analysis, suggesting significant activation of the pathway in these cases. In contrast, CRC_P09 is characterised by higher scores in MAPK, EGFR, TGFα, and NFκB pathways, indicating a pronounced activation of these pathways in this particular sample. This differential pathway activity underscores the heterogeneity of molecular signatures within the patient cohort and may have implications for therapeutic strategies targeting these pathways. Within the normal epithelial cell sub-clusters, we found that the stem-like/transit-amplifying sub-cluster exhibited the highest stemness. In contrast, most tumour cell subpopulations had higher differentiation potential scores than the stem-like/transitionally proliferative cell subclusters, except for the CRC_P08T and CRC_P10T tumour cell subclusters. This finding aligns with the majority of CRC cells believed to originate from stem cells or cells with stem-like characteristics [54], [55], [56].

#### 3.2.4 Gastric cancer

Similarly, subclusters distinct from GC exhibited highly expressed cancer-related genes and key regulatory transcription factors (Fig. S16A-B, Tables S3 and S5). For instance, the tumour cell subcluster GC_P02T exhibited a high expression of MSLN, a cancer-associated antigen which is upregulated in various malignant tumours, including GC [57]. ONECUT2, a pivotal TF in GC_P08T_1 that accelerates tumourigenesis by activating the expression of ROCK1 in GC [58]. In our analysis of patient GC_P02, we detected notable abnormal amplifications on chromosomes 16 and 20. Interestingly, many highly expressed sub-cluster-specific DEGs, including NPW, MSLN, WFDC2, COL9A3, and LAMA5 were located in these CNV regions. Further investigation of the CNV scores for these genes revealed a significant increase, indicating that the elevated expression of these genes was likely due to variations in copy number. Other biological differences in these sub-clusters are shown in Fig. S12 and Fig. S16. CytoTRACE results indicated that the GC_P08T_1 and GC_P08T_2 tumour cell subpopulations had higher differentiation potential scores. This is consistent with the PROGENy and GSVA results (Fig. S16C, Fig. S21), where the GC_P08T_1 and GC_P08T_2 tumour cell subpopulations were predominantly enriched in the hedgehog signalling pathway and in DNA repair and G2/M checkpoint signalling pathways, respectively.

#### 3.2.5 Hepatocellular carcinoma

Similar to other types of cancer, there was a high degree of heterogeneity in the DEGs and key TFs across various subclusters of HCC (Fig. S17A-B). For instance, PITX2, the primary regulatory transcription factor of the HCC_P02T sub-cluster, has been shown to increase the stemness characteristics of liver cancer cells by upregulating key developmental factors in liver progenitor cells [59], which aligns with its higher stemness (Fig. S17D). Similarly, in HCC_01, we observed amplification of chromosome 1 (Fig. S13), and S100A9, the DEG of HCC_P01T, was located at this position. The GGH gene, which is highly expressed in HCC_P04T and located on chromosome 8, was amplified in patient HCC_P04. Fig. S17 illustrates the biological differences between the various tumour cell subclusters in hepatocellular carcinoma. There is significant heterogeneity in the activity of VEGF, MAPK, EGFR, TGF-β, and hypoxia signalling across different tumour cell subclusters (Fig. S17C). Notably, the HCC_P02T, HCC_P01T and HCC_04T tumour cell subcluster exhibits heightened activity in the Wnt pathway, while the HCC_P08T tumour cell subpopulation shows increased activity in the TGF-β, EGFR, MAPK, and NFκB pathway, aligning with the results obtained during CytoTRACE analysis (Fig. S17D).

In addition to heterogeneity, biological characteristics in various tumour cell sub-clusters showed similarities within the same cancer type and even among different cancers. For example, high activity of TFs belonging to the human forkhead-box (FOX) gene family has been observed in many subclusters. FOXA1 has been reported as a key TF in LUAD_P01T, and FOXC1 is a key TF in LUAD_P04T; similarly, FOXJ2 in LUAD_P07T_2, FOXK2 in LUAD_P09T, FOXC2 and FOXQ1 in BC_P02T, FOXI1 in CRC_P07T, FOXN4 and FOXO1 in HCC_P03T, and FOXO4 in HCC_P04T. Researchers have found that dysregulated gene expression of the FOX family leads to diseases such as congenital disorders, diabetes mellitus, or carcinogenesis [60]. Additionally, TFs of the E2F family, known for their multifaceted roles in transcriptional activation and repression, regulation of cell proliferation and apoptosis, modulation of tumour suppression, and oncogenesis, have been identified in various subclusters [61], [62], specifically LUAD_P01T, LUAD_04T, LUAD_07T_1, BC_P02T, CRC_07T, and HCC_P01T. Interestingly, although the tumour cell subclusters showed distinct genomic and transcriptomic variations, some subclusters had similar oncogenic signalling pathway activities. We found that LUAD_P05T tumour cells were more active in the trial pathway. In addition to LUAD_P05T, there were also other subclusters exhibiting similar characteristics, like LUAD_P01T, BC_P06T, CRC_P02T, CRC_P08T, CRC_P10T, HCC_P04T, and HCC_P05T, although they also show activity in other pathways. The LUAD_P06T, HCC_P08T, CRC_P09T, GC_P02T, and BC_P02T tumour cell subclusters, on the other hand, were more active in the EGFR, MAPK, NFκB, and TNF-α pathways. In contrast, other subclusters, including LUAD_P04T, LUAD_P09T, BC_P05T, CRC_P07T, and HCC_P02T, showed much lower activity in these pathways but higher activity in the Wnt pathway. The CytoTRACE results showed that the majority of tumour cell subclusters had a higher stemness than normal epithelial cell types, while some tumour cell subclusters a had higher stemness than others (Fig. 3D and Figs. S14-S17D). For example, in LUAD, the cancer cell subclusters from patients LUAD_P04T, LUAD_P07T, and LUAD_P09T exhibited high stemness levels, possessing high potential for self-renewal and multilineage differentiation, whereas the other subclusters had lower stemness levels (Fig. 3D). We propose that the consistency in the activation of these [21] oncogenic pathway could serve as a foundation for classifying cancers for clinical treatment.

### 3.3 Specific biomarker screening for tumour cell subtypes

**Fig. 4:**
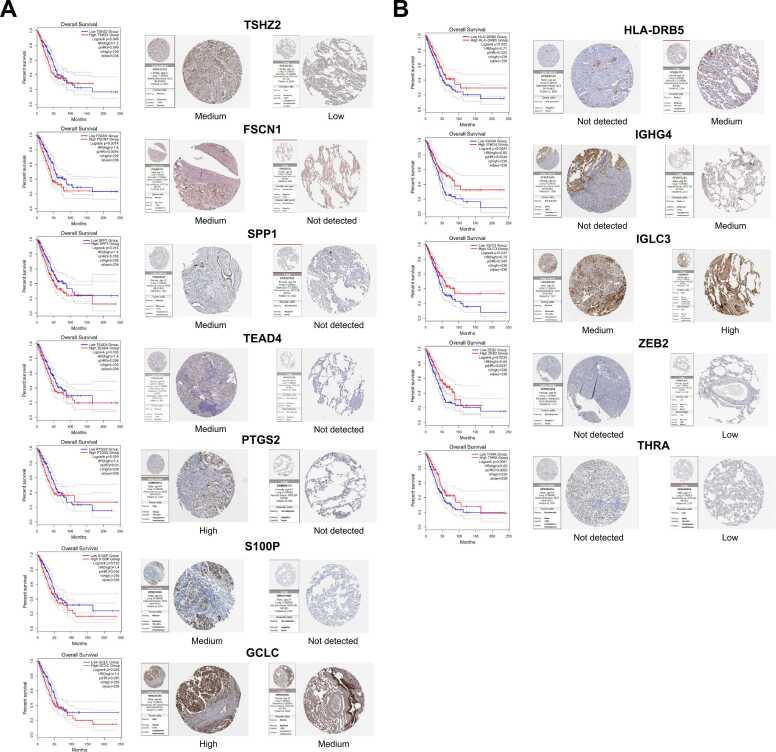
Specific biomarkers of tumour cell subtypes from lung adenocarcinoma. The biomarkers were classified into two groups based on the prognosis results of survival analysis. (A) Biomarkers with poorer prognosis. (B) Biomarkers with better prognosis. For each biomarker, the left image is the Kaplan-Meier curve plot, downloaded from the GEPIA2 database. In survival analysis, a higher curve indicates this group has a higher survival rate, suggesting that high or low expression of this biomarker is beneficial for cancer patients’ survival. Conversely, a lower curve signifies a lower survival rate. For each biomarker, the middle and right images were sourced from the HPA database, representing the protein expression of the biomarker in tumour tissue samples and normal tissue samples, respectively. See supplementary materials for the other four cancer types.

To screen biomarkers for tumour cell subclusters that can significantly distinguish the survival of patients, we input highly expressed DEGs and key TFs of tumour cell subclusters into the survival analysis function module of the GEPIA2 database [40] to draw Kaplan-Meier curves. Subsequently, the above markers were verified using the HPA database [41], and genes whose survival analysis results were consistent with the protein expression levels in the database were regarded as potential biomarkers for tumour cell subclusters. We systematically classified the identified biomarkers into two distinct groups based on their expression levels and associated prognostic implications. The first group was comprised of biomarkers with higher expression levels that were indicative of a poorer prognosis (Fig. 4A, Figs. S23-S26A), whereas the second group included biomarkers with higher expression levels associated with a more favourable prognosis (Fig. 4B, Figs. S23–S26B). To further validate the efficacy and applicability of the identified biomarkers, we conducted external validation on independent datasets Kaplan-Meier Plotter [42], [43], [44] and PanCanSurvPlot [45] (Figs. S27−S31). Finally, the tumour cell subcluster biomarkers of the five cancers were identified and are listed in Table S7.

#### 3.3.1 Specific biomarkers of lung adenocarcinoma

Fig. 4 shows 12 potential biomarkers of LUAD. We categorised these biomarkers into two groups based on the prognostic results of survival analysis. Patients with a high expression of TSHZ2, SPP1, FSCN1, and TEAD4 had a poorer prognosis (Fig. 4A), whereas those with a high expression of DRB5, ZEB2, IGHG4, and IGLC3 had a better prognosis (Fig. 4B). In particular, high expression of the three biomarkers identified in patient LUAD_P04, namely SPP1 and FSCN1, indicated a poorer prognosis. As shown in the previous section, the results from the CytoTRACE and GSVA analyses indicated that the LUAD_P04T subcluster possesses characteristics of cancer stem cells (CSCs) and exhibits higher stemness. As higher stemness of tumour cells is often related to worse prognosis [63], [64], [65], these three genes have the potential to serve as biomarkers for lung cancer patients with cancer stem cells and poor prognosis [66], [67], [68], [69]. The upregulation of TEAD4, a specific biomarker of LUAD_P05, may lead to excessive transcription and phosphorylation of the ERK protein, thereby accelerating the progression of tumour development and resulting in a poor prognosis. Therefore, it is a promising therapeutic target [70].

#### 3.3.2 Specific biomarkers of breast cancer

Potential biomarkers from various tumour cell subclusters in other cancers are shown in Figs. S23−S26. The specific biomarkers after validation of the external database for each BC tumour subcluster are presented in Fig. S28 and Table S7, where we identified some biomarkers that are confirmed to be associated with cancer and could potentially serve as prognostic markers. Consistent with the survival curves, where patients with a low expression of KRT15 have a shorter survival span, KRT15, a specific biomarker of the BC_P06T subcluster, was significantly associated with advanced clinical pathological factors and unfavourable overall survival (OS). It may serve as a promising prognostic marker for the diagnosis and analysis of patients with breast invasive carcinoma (BRCA) [71]. Moreover, CALML3, a biomarker of BC_P05T, may function as a tumour suppressor gene, offering an early warning value for pulmonary metastasis of liver cancer. It has the potential to serve as an early diagnostic marker for lung metastasis in liver cancer and as a new target for inhibiting the growth and spread of liver cancer [72].

#### 3.3.3 Specific biomarkers of colorectal cancer

ANKRD22, a specific biomarker from the CRC_P01T subcluster (Fig. S29), activates the Wnt/β-catenin pathway by means of regulating the expression of NuSAP1 [73], which is consistent with the high activity of the Wnt pathway and high stemness in subcluster CRC_P01T (Fig. S15C-D). It has potential as a biomarker of tumour cell subclusters with stem cell-like properties. Consistent with the survival curves of GFI1 in Fig. S29, Chen et al. demonstrated that GFI1 functions as a tumour suppressor gene in CRC, with low expression levels of GFI1 promoting the development of malignant colon tumours [74]. Additionally, in a study by Zhu *et al.*, HOXC8, a specific biomarker of the CRC_P03T sub-cluster (Fig. S29), was significantly overexpressed in CRC samples compared to normal samples and was notably associated with invasion-related pathways, especially EMT [75]. This is consistent with the GSVA results showing that the highly expressed genes in CRC_P03T were enriched in the EMT pathway (Fig. S20).

#### 3.3.4 Specific biomarkers of gastric cancer

Following the validation across the aforementioned datasets, we have identified two GC-specific biomarkers, namely RARB from GC_P02T and SNCG from GC_P07T (Table. S7, Fig. S30). SNCG is a specific biomarker of GC_P07T (Fig. S30), whose expression levels in gastric cancer cells were significantly elevated compared to those in normal cells. Pan *et al*. revealed that SNCG is correlated with tumour lymph node metastasis stage and tumour size, which are of great significance for the diagnosis and prognosis of GC [76]. By targeting RARB, a specific biomarker of the GC_P02T subcluster, it is possible to modulate the MAPK signalling pathway, which in turn can influence the apoptotic and differentiation processes of cancer cells [77]. This strategic intervention holds promise for impacting the functional behaviour of cancer cells, potentially leading to novel therapeutic approaches in the field of oncology.

#### 3.3.5 Specific biomarkers of hepatocellular carcinoma

Similarly, we employed the aforementioned analytical process to screen and validate specific biomarkers for various cell subclusters in patients with HCC. We identified two relatively reliable biomarkers: a specific biomarker for HCC_P03T and CTTN and one for HCC_P04T and BHMT. CTTN has been shown to promote cancer development owing to its elevated expression, which is indicative of a poor prognosis. It has been validated as a novel biomarker of cancer and has been identified as a potential therapeutic target for drug development [78]. Jin *et al.* demonstrated that downregulation of BHMT in HCC is associated with poor prognosis, a finding that corroborates the survival curves plotted using the GEPIA database. Specifically, high BHMT patient in HCC_P04 may serve as a positive indicator of a better prognosis [79].

### 3.4 Specific drug screening for tumour cell subtypes

Based on the biological differences among tumour cell sub-clusters, we believe that specific drug treatments are needed to reverse various pathological changes. CORN is a database of condition-based (natural compound/small molecule/drug treatments and gene perturbations) TRSNs [33]. It contains an online TRSN-matching tool that allows users to identify drugs or potential therapeutics targeting specific diseases by matching drug-induced TRSNs and disease transcriptomic changes. The higher the matching score, the more similar are the drug-induced TRSNs and input disease transcriptomic changes. A positive score for the matched drug-induced TRSN indicates that the reported drug has the potential to reverse the input gene expression changes in the disease. Therefore, to identify treatments specific to tumour cell sub-clusters, we input DEGs between tumour cells from each sub-cluster and all normal epithelial cells of the corresponding cancer species into the CORN matching tool. As mentioned above, searching for condition-oriented TRSNs with positive scores would be helpful for seeking specific treatments for each tumour cell subcluster. The results are summarised in Table S8.

We discovered that different subclusters of tumour cells within the same cancer type could match with the same drug or condition (Table S8). The tumour cell clusters LUAD_P04T, LUAD_P06T, LUAD_P07T_1, and LUAD_P07T_2 matched tanespimycin, also known as 17-AAG, a potent inhibitor of heat shock protein 90 (HSP90). Tanespimycin exerts its effects by inhibiting HSP90 along with the downstream Wnt signalling pathway, thereby suppressing the self-renewal and invasion of tumour cells from patients LUAD_P04, LUAD_P06, and LUAD_P07 [80]. This finding aligns with the CytoTRACE analysis results showing that these three tumour cell sub-clusters showed high stemness (Fig. 3D). In addition, several tumour cell subpopulations were identified across various cancer types are matched with perhexiline, such as LUAD_P04T, LUAD_P07T_2, LUAD_P09T, CRC_P03T, and GC_P08T_1 (Table S8). Interestingly, these tumour cell subpopulations all exhibit activation of the Wnt pathway, while showing lower activity in the EGFR, MAPK, NFκB, or TNF-α pathway. This indicates that tumour subpopulations with similar characteristics and biological functions may be sensitive to the same or similar drugs.

In contrast, different subclusters of the same cancer type matched subcluster-specific drugs, suggesting the possibility of using precision medicine (Table S8). For example, the lung tumour cell subcluster LUAD_P04T was specifically matched to vorinostat, a histone deacetylase (HDAC) inhibitor that can block cancer cell proliferation both in *vitro* and *vivo*, and has been approved by the U.S. Food and Drug Administration for the treatment of cutaneous T-cell lymphoma [81]. The anti-proliferative effect of vorinostat is believed to result from the inhibition of HDAC activity, which leads to the accumulation of acetylated proteins, including histones. Vorinostat may also promote the acetylation of numerous TFs, including E2F1, resulting in altered expression of downstream genes [82], [83]. This finding aligns with the GSVA and SCENIC results showing that LUAD_P04T displayed significant enrichment of DEGs, primarily in the E2F target pathway, and E2F1 was identified as one of its key TFs with a robust regulatory relationship. Based on the matching results, a combination of tanespimycin and vorinostat was recommended for LUAD_P04. A previous study reported that the combination of an HDAC and HSP90 inhibitor could induce more apoptosis in cancer cells than treatment with either agent alone [84]. The lung tumour cell cluster of LUAD_P06 was specifically matched to PD-0325901, an oral MAPK/ERK kinase inhibitor. PD-0325901 prevented the phosphorylation and subsequent activation of MAPK, which was significantly activated in LUAD_P06T [85]. Furthermore, we discovered that even tumour cell subclusters derived from the same patient could match different therapeutic drugs. For instance, the tumour cell subpopulation GC_P08T_1 matched NVP-AEW541, whereas GC_P08T_2 matched wortmannin, which revealed intratumor heterogeneity within the same patient and the necessity for combination therapies involving multiple drugs. This was probably because NVP-AEW541 has been found to significantly reduce tumour growth, vascularisation, and VEGF expression [86]. Compared with GC_P08T_2, GC_P08T_1 exhibited significant activation of the VEGF pathway (Fig. S21C). Meanwhile, wortmannin exerted its effects by inhibiting the upregulation of the E2F pathway in GC_P08T_2 (Fig. S21C) [87].

## 4 Discussion

In traditional clinical and histopathological cancer classifications, guidance provided by morphological features, such as imaging and microscopic cell morphology, is limited. However, it fails to comprehensively reflect the biological aspects of tumour behaviour and individual differences in recurrence, metastasis, and sensitivity to radiation or chemotherapy. Although scRNA-seq technology has made tremendous progress in its oncology application, significantly advancing cancer-related research, most studies currently focus on the tumour microenvironment, with limited research specifically targeting cancer epithelial cells, especially in the five most common epithelial-derived cancers: LUAD, BC, CRC, GC, and HCC. Also, the efficient and rapid application of this technology in clinical settings, achieving high accuracy with minimal resolution, such as in the personalized treatment of tumour heterogeneity as discussed in this paper, remains a challenging issue. Hence, in this study, we analysed the scRNA-seq data from these five cancers to explore tumour heterogeneity, identify biomarkers and drugs for individual patients, and provide a general direction for clinical cancer classification.

Both inter- and intratumoral heterogeneities were identified when the tumour cells were classified into subclusters. (Fig. 2C). Tumour cell heterogeneity was observed at both the genomic and transcriptomic levels. CNVs, highly expressed genes, and key TFs activated in tumour cells were found to have specific patterns in different tumour cell subclusters, even within the same type of cancer. However, interestingly, under diverse genomic variation and expression profiles, some tumour cell subclusters share similar biological functions and phenotypes. In all five cancer types, the tumour cell sub-clusters of many patients exhibited higher levels of stemness (Fig. 3D, Figs. S4-S17D), which was correlated with the activation of the Wnt pathway in the PROGENy analysis (Fig. 3C, Figs. S14−S17C). Conversely, a subset of tumour cell subclusters with higher levels of differentiation showed activation of the EGFR, MAPK, and Trail pathways (Fig. 3C, Figs. S14−S17C). This scenario may be attributed to the parallel but convergent evolution of tumour cells derived from many different genomic variations to adapt to a few different types of microenvironments.

**Fig. 5:**
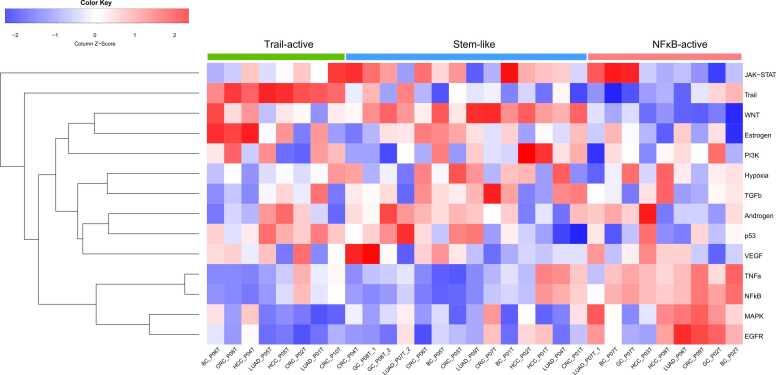
Heatmap of PROGENy pathway activity in each tumour cell subpopulation across five cancer types. All tumour cell subpopulations were divided into three categories and marked with different colours: TRAIL-active type in green, stem-like type in blue, and NFκB-active type in red, respectively.

To uncover the potential patterns of stemness and pathway enrichment among tumour cells in different types of cancer, we compiled the activity profiles of distinct tumour cell subpopulations across 14 oncogenic signalling pathways in five different types of cancer (Fig. 5). Fig. 5 indicates that all tumour subclusters can be categorised into three major groups: stem-like subclusters (a group with high stemness levels), NFκB-active subclusters (a group with high activity of the NFκB and its related pathways), and Trail-active subclusters (a group with high activity of the Trail pathway). Tumour cell sub-clusters with high stemness, such as LUAD_P04T, LUAD_P09T, BC_P05T, CRC_P07T, and HCC_P02T, displayed high activity in the Wnt signalling pathway. Previous studies have confirmed that the Wnt pathway plays a crucial regulatory role in supporting the maintenance and survival of CSCs and is thus currently recognised as one of the main targets for anti-CSC therapy [88]. The Wnt pathway is also highly conserved in tumour evolution, which corresponds to the results of many subpopulations in our study that showed enrichment in this pathway. On the other hand, tumour cell subpopulations including LUAD_P06T, BC_P02T, BC_P07T, CRC_P09T, GC_P02T, GC_P07T, HCC_P03T, and HCC_P08T exhibit higher differentiation levels and the activation of the EGFR, MAPK, NFκB, and TNF-α pathways. The NFκB signalling pathway is their key pathway. The TNF-α activates the NFκB and MAPK signalling pathways through TRAF2, thereby promoting inflammation and cell survival and proliferation [89], [90]. Simultaneously, EGF can activate NFκB through the IKK complex via distinct pathways [91]. Subsequently, the nuclear translocation of NFκB p65 can induce the transcription of several genes involved in EMT induction, enhancing the proliferation and metastasis of tumour cells [92]. Moreover, NF-κB/EMT axis is involved in mediating drug resistance in tumour cells. Trial-active groups, such as LUAD_P01T, LUAD_P05T, BC_P06T, CRC_P02T, CRC_P08T, CRC_P10T, HCC_P04T, and HCC_P05T, showed high activity in the trial-active pathway. The trial pathway has been found to activate the PI3K pathway, thereby inducing cell proliferation [93], although its main function is to induce apoptosis. This may render this group of subclusters sensitive to apoptosis-inducing drugs, leading to a better prognosis for patients. In comparison, the Wnt signalling is crucial for development and tissue regeneration, whereas NF-κB is a key master of inflammation. The Wnt and NFκB signalling pathways regulate, through independent cascades, the expression of different subsets of target genes controlling cell proliferation. Recent findings suggest that these two signalling pathways may cross-regulate each other, creating a complex regulatory network. So far, evidence supports that Wnt signalling downregulates production of proinflammatory cytokines, including IL-1β, IL-6, IL-8, and TNF-α [94], [95]. Besides, NF-κB has been shown to indirectly regulate the Wnt pathway through regulation of target genes that affect β-catenin activity or stability [96]. In addition, the TRAIL pathway mainly participates in tumour therapy by inducing apoptosis. Altogether, stem-like subclusters may display lower proliferation and invasiveness, but could potentially possess high stem cell characteristics and self-renewal capability; NFκB-active subpopulations may exhibit higher level of inflammatory response, proliferation, and invasiveness, associated with the promoting effects of NFκB; TRAIL-active subpopulations possibly exhibit an increased sensitivity to apoptosis. These biological, functional, and characteristic differences result in the sensitivity of these three categories of tumour subpopulations to different treatments and drugs.

Biomarkers identified in the tumour cell subclusters also reflected the characteristics of the three groups of subclusters. For example, the specific biomarker of the LUAD_P04T sub-cluster, FSCN1, activates the Wnt pathway, which is consistent with the high activity of LUAD_P04T in the Wnt pathway in the PROGENy analysis, and its high expression suggests a poor prognosis [97]. According to our findings, the TRIAL-active-type tumour cell subclusters typically align with biomarkers linked to better prognosis, such as HLA-DRB5 of LUAD_P01T and BHMT of HCC_P04T (Fig. 4B, Fig. S31B), whereas the prognosis for the stem-like and NFκB-active type may be complex due to stem cell characteristics, abnormal pathway activation, or treatment resistance.

Subsequently, we identified patient-specific drugs, as listed in Table S8. Different subclusters of tumour cells, even from different cancers, could match the same drug; however, many tumour cell subclusters derived from the same cancer type or even the same patient could match different therapeutic drugs. Interestingly, tumour cell subclusters from the same group, classified as mentioned above, tended to match the same or similar drugs with similar pharmacological mechanisms. For instance, in the case of stem-like cancer cells, we found that the drugs perhexiline and withaferin exhibited promising therapeutic potential. The in *vivo* anti-tumour potential of perhexiline has been investigated in multiple cancer types in mice, including BC [98], CRC [99], GC [99], glioblastoma [100], liver cancer [101], and T-cell acute lymphoblastic leukaemia [102]. Perhexiline may exert its effects by inhibiting FYN, which can phosphorylate β-catenin, releasing it from the junctional complex to stimulate WNT target gene expression. Therefore, perhexiline is recommended as a broad-spectrum anti-tumour drug for the stem-like type tumours defined in our study. We also screened for drugs targeting trial-active tumour cells, such as BRD-K61102114 and chelerythrine, and drugs targeting NFκB-active tumour cells, such as mitoxantrone. The antitumor effects of chelerythrine have been recognised, and it has been demonstrated to overcome the resistance of leukaemia KG1a cells to TRAIL-induced apoptosis [103] and induce apoptosis in certain tumour cells [104], [105]. Researchers have confirmed the inhibitory effect of mitoxantrone on NF-κB pathway activation and its ability to reduce the secretion of TNF-α. These findings are consistent with the results of our drug screening for NF-κB active tumours, thereby validating the effectiveness of our methodology to a certain extent [106].

**Fig. 6:**
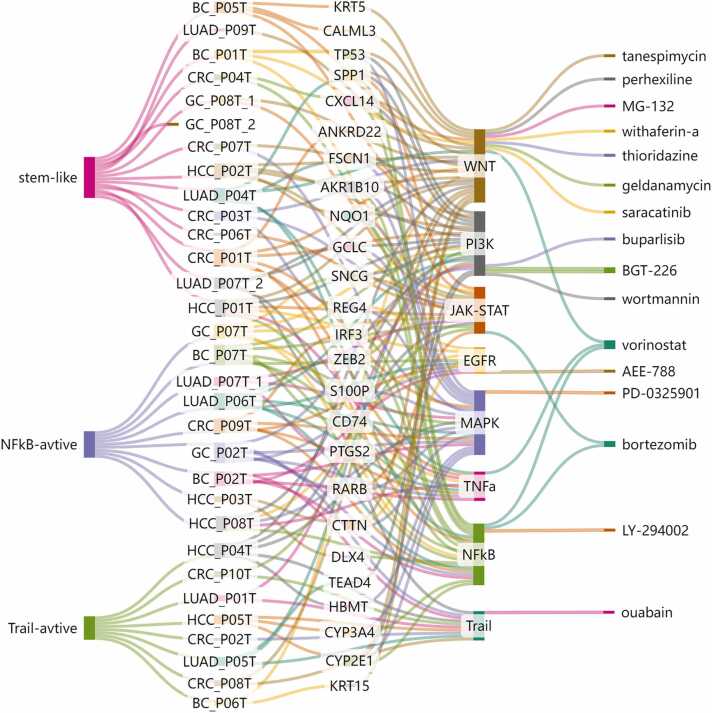
The connections between groups of tumour cell subpopulations, tumour cell subpopulations from different patient sources, identified biomarkers, enriched PROGENy pathways, and matched drugs.

Finally, a Sankey diagram was created to explore the relationships between different tumour cell subclusters, biomarkers, oncogenic signalling pathways, and potential therapeutic drugs in various types of cancer (Fig. 6). Through our comprehensive analysis, we successfully explored intertumoral and intratumoral heterogeneity by examining variations in genomic levels, gene expression patterns, biological functions, specific biomarkers, and drugs among different tumour cell subclusters. Even within the same patient, there could be two distinct tumour sub-clusters, reflecting the complex evolutionary outcomes of the tumours. However, as shown in Fig. 6, all tumour cell sub-clusters across the five cancer types could be categorised into three major groups based on their behavioural trends. This indicates that we should not only focus on differences but also on similarities among patients, enabling us to more accurately and quickly determine the most suitable treatment options for each type of tumour and patient.

Many outstanding single-cell studies have focused on the tumour immune microenvironment, which investigates how cells and molecules within it influence tumour progression, immune evasion, and therapeutic resistance, as well as the heterogeneity of different tumour microenvironments and the development of new immunotherapies [16], [17], [18], [19]. There also have been some studies on epithelial tumour cells, they mostly focus on exploring EMT and cell trajectories within a single type of tumour [^111–113^]. Researchers such as Zhang et al. and Gao et al. have also proposed classifications for epithelial tumours; however, specific treatment plans for each type remain unclear [20], [21], [22]. Our particular focus was on the sub-clusters of epithelial tumour cells, examining their varying degrees of malignancy, the distinct signalling pathways they employ, their capabilities for invasion and metastasis, and their potential for differentiation. We leveraged these biological differences to explore the potential prognostic disparities and individual or subtype-specific treatments that may arise from them. As mentioned above, we hope that this analytical framework can facilitate an efficient and rapid precision medicine approach to single-cell data in clinical settings, enabling a transition from a 'one-size-fits-all' treatment to a 'tailored medicine for each patient' paradigm.

Although we have arrived at some meaningful findings and results, we would like to point out the limitations of this study. First, the sample sizes for each cancer type were relatively small. We must acknowledge that owing to the limitations in sample size, the findings may not be widely applicable to a broader range of diverse patient populations. In the future, we endeavour to address this limitation by incorporating larger and more diverse cohorts to further enhance the robustness and applicability of the study outcomes. Second, in this study, we mainly focused on epithelial cells but did not explore the effects of other cell types, such as immune and stromal cells, on tumour cell heterogeneity. Third, limitations of scRNA-seq technology, such as high dropout rates and potential biases, may have affected the accuracy of our analyses. Incorporating different types of data, such as bulk transcriptome and single-cell multiomics data, may provide further validation and a deeper understanding of the mechanisms involved in tumour heterogeneity. Finally, as the majority of our samples were acquired through single-region sampling and considering the limited heterogeneity among tumour cells within neighbouring areas, only two instances of intratumor heterogeneity were identified in our study. Consequently, our research primarily focused on intertumoral heterogeneity, with less emphasis on intratumoral heterogeneity.

## 5 Conclusions

In conclusion, by analysing single-cell transcriptomic data from five common types of cancer, we investigated the heterogeneity of tumour cells and the underlying biological differences at the single-cell level and provided a potential tumour cell subcluster classification which could be referenced in clinical settings. This study demonstrated the promising prospects of scRNA-seq technology for transitioning from traditional morphological classification to a more precise molecular classification. By analysing tumour cell subcluster variations at the genome, transcriptome, transcriptional regulation, signalling pathway, and stemness level, we found that tumour cell subclusters from different cancers varying in genome, transcriptional regulation and transcriptome could converge into three major groups: stem-like subclusters (a group with high stemness levels), NFκB-active subclusters (a group with high activity of the NFκB and its related pathways), and TRAIL-active subclusters (a group with high activity of the TRAIL pathway), which exhibit similar activated signalling pathway patterns and tumour cell stemness phenotypes within each group. Furthermore, by integrating multiple bioinformatics databases, we identified specific biomarkers and potential therapeutic drugs for each tumour cell subcluster and different subcluster groups. These discoveries emphasise the need to transition towards a personalised treatment paradigm that recognises the concept of ‘same disease, different treatments’ as well as ‘different patients, similar treatments’, providing new insights for personalised therapy.

## CRediT authorship contribution statement

**Xinying Zhang:** Writing – review & editing, Writing – original draft, Visualization. **Zixin Yang:** Methodology, Data curation, Conceptualization. **Jiajie Xie:** Writing – original draft, Visualization, Investigation. **Yaohua Hu:** Writing – review & editing, Supervision, Funding acquisition, Conceptualization. **Carisa Kwok Wai Yu:** Writing – review & editing, Supervision. **Jing Qin:** Writing – review & editing, Supervision, Project administration, Conceptualization.

## Declaration of Competing Interest

The authors declare that they have no known competing financial interests or personal relationships that could have appeared to influence the work reported in this paper.

## Data Availability

Raw and processed scRNA-seq datasets are available for download in NCBI GEO with the following accession numbers: GSE131907 for lung adenocarcinoma (LUAD), GSE161529 for breast cancer (BC), GSE132465 for colorectal cancer (CRC) and GSE149614 for hepatocellular carcinoma (HCC). Additionally, scRNA-seq dataset for gastric cancer (GC) is under the accession number of OMIX001073 in the OMIX database.

## References

[1] Bray F., Laversanne M., Sung H. (2024). Global cancer statistics 2022: GLOBOCAN estimates of incidence and mortality worldwide for 36 cancers in 185 countries. CA Cancer J Clin.

[2] Dagogo-Jack I., Shaw A.T. (2018). Tumour heterogeneity and resistance to cancer therapies. Nat Rev Clin Oncol.

[3] Jerby L., Wolf L., Denkert C. (2012). Metabolic associations of reduced proliferation and oxidative stress in advanced breast cancer. Cancer Res.

[4] Graf J.F., Zavodszky M.I. (2017). Characterizing the heterogeneity of tumor tissues from spatially resolved molecular measures. PLoS One.

[5] Zhang S., Dong P., Pan Z. (2023). Comparison of gene mutation profile in different lung adenocarcinoma subtypes by targeted next-generation sequencing. Med Oncol.

[6] Zhang J., Spath S.S., Marjani S.L., Zhang W., Pan X. (2018). Characterization of cancer genomic heterogeneity by next-generation sequencing advances precision medicine in cancer treatment. Precis Clin Med.

[7] Zhang X. (2023). Molecular Classification of Breast Cancer: Relevance and Challenges. Arch Pathol Lab Med.

[8] Punt C.J., Koopman M., Vermeulen L. (2017). From tumour heterogeneity to advances in precision treatment of colorectal cancer. Nat Rev Clin Oncol.

[9] The Cancer Genome Atlas Research Network (2014). Comprehensive molecular characterization of gastric adenocarcinoma. Nature.

[10] Roma-Rodrigues C., Mendes R., Baptista P.V., Fernandes A.R. (2019). Targeting tumor microenvironment for cancer therapy. Int J Mol Sci.

[11] Dai W., Zhou F., Tang D. (2019). Single-cell transcriptional profiling reveals the heterogenicity in colorectal cancer. Med (Baltim).

[12] Chung W., Eum H.H., Lee H.O. (2017). Single-cell RNA-seq enables comprehensive tumour and immune cell profiling in primary breast cancer. Nat Commun.

[13] Wu F., Fan J., He Y. (2021). Single-cell profiling of tumor heterogeneity and the microenvironment in advanced non-small cell lung cancer. Nat Commun.

[14] Qian J., Olbrecht S., Boeckx B. (2020). A pan-cancer blueprint of the heterogeneous tumor microenvironment revealed by single-cell profiling. Cell Res.

[15] Ren X., Zhang L., Zhang Y. (2021). Insights Gained from Single-Cell Analysis of Immune Cells in the Tumor Microenvironment. Annu Rev Immunol.

[16] Zheng L., Qin S., Si W. (2021). Pan-cancer single-cell landscape of tumor-infiltrating T cells. Science.

[17] Yang Y., Chen X., Pan J. (2024). Pan-cancer single-cell dissection reveals phenotypically distinct B cell subtypes. Cell.

[18] Cheng S., Li Z., Gao R. (2021). A pan-cancer single-cell transcriptional atlas of tumor infiltrating myeloid cells. Cell.

[19] Tang F., Li J., Qi L. (2023). A pan-cancer single-cell panorama of human natural killer cells. Cell.

[20] Zhang M., Hu S., Min M. (2021). Dissecting transcriptional heterogeneity in primary gastric adenocarcinoma by single cell RNA sequencing. Gut.

[21] Guo D.Z., Zhang X., Zhang S.Q. (2024). Single-cell tumor heterogeneity landscape of hepatocellular carcinoma: unraveling the pro-metastatic subtype and its interaction loop with fibroblasts. Mol Cancer.

[22] Kinker G.S., Greenwald A.C., Tal R. (2020). Pan-cancer single-cell RNA-seq identifies recurring programs of cellular heterogeneity. Nat Genet.

[23] Barrett T., Troup D.B., Wilhite S.E. (2009). NCBI GEO: archive for high-throughput functional genomic data. Nucleic Acids Res.

[24] Hao Y., Hao S., Andersen-Nissen E. (2021). Integrated analysis of multimodal single-cell data. Cell.

[25] Korsunsky I., Millard N., Fan J. (2019). Fast, sensitive and accurate integration of single-cell data with Harmony. Nat Methods.

[26] Lambrechts D., Wauters E., Boeckx B. (2018). Phenotype molding of stromal cells in the lung tumor microenvironment. Nat Med.

[27] Schiller H.B., Montoro D.T., Simon L.M. (2019). The Human Lung Cell Atlas: A High-Resolution Reference Map of the Human Lung in Health and Disease. Am J Respir Cell Mol Biol.

[28] The Tabula Muris Consortium (2018). Overall coordination., Logistical coordination. *et al.* Single-cell transcriptomics of 20 mouse organs creates a *Tabula Muris*. Nature.

[29] Treutlein B., Brownfield D.G., Wu A.R. (2014). Reconstructing lineage hierarchies of the distal lung epithelium using single-cell RNA-seq. Nature.

[30] Puram S.V., Tirosh I., Parikh A.S. (2017). Single-cell transcriptomic analysis of primary and metastatic tumor ecosystems in head and neck cancer. Cell.

[31] Peng J., Sun B.F., Chen C.Y. (2019). Single-cell RNA-seq highlights intra-tumoral heterogeneity and malignant progression in pancreatic ductal adenocarcinoma. Cell Res.

[32] Finak G., McDavid A., Yajima M. (2015). MAST: a flexible statistical framework for assessing transcriptional changes and characterizing heterogeneity in single-cell RNA sequencing data. Genome Biol.

[33] Leung R., Jiang X., Zong X. (2022). CORN-Condition Orientated Regulatory Networks: bridging conditions to gene networks. Brief Bioinform.

[34] Aibar S., Gonzalez-Blas C.B., Moerman T. (2017). SCENIC: single-cell regulatory network inference and clustering. Nat Methods.

[35] Van de Sande B., Flerin C., Davie K. (2020). A scalable SCENIC workflow for single-cell gene regulatory network analysis. Nat Protoc.

[36] Sondka Z., Dhir N.B., Carvalho-Silva D. (2024). COSMIC: a curated database of somatic variants and clinical data for cancer. Nucleic Acids Res.

[37] Hanzelmann S., Castelo R., Guinney J. (2013). GSVA: gene set variation analysis for microarray and RNA-seq data. BMC Bioinforma.

[38] Schubert M., Klinger B., Klunemann M. (2018). Perturbation-response genes reveal signaling footprints in cancer gene expression. Nat Commun.

[39] Gulati G.S., Sikandar S.S., Wesche D.J. (2020). Single-cell transcriptional diversity is a hallmark of developmental potential. Science.

[40] Tang Z., Kang B., Li C., Chen T., Zhang Z. (2019). GEPIA2: an enhanced web server for large-scale expression profiling and interactive analysis. Nucleic Acids Res.

[41] Uhlen M., Fagerberg L., Hallstrom B.M. (2015). Proteomics. Tissue-based map of the human proteome. Science.

[42] Gyorffy B. (2024). Transcriptome-level discovery of survival-associated biomarkers and therapy targets in non-small-cell lung cancer. Br J Pharm.

[43] Gyorffy B. (2021). Survival analysis across the entire transcriptome identifies biomarkers with the highest prognostic power in breast cancer. Comput Struct Biotechnol J.

[44] Gyorffy B. (2024). Integrated analysis of public datasets for the discovery and validation of survival-associated genes in solid tumors. Innov (Camb).

[45] Lin A., Yang H., Shi Y. (2022). PanCanSurvPlot: a large-scale pan-cancer survival analysis web application. bioRxiv.

[46] Han Z.W., Lyv Z.W., Cui B. (2020). The old CEACAMs find their new role in tumor immunotherapy. Invest N Drugs.

[47] Beauchemin N., Arabzadeh A. (2013). Carcinoembryonic antigen-related cell adhesion molecules (CEACAMs) in cancer progression and metastasis. Cancer Metastas-- Rev.

[48] Cheng W.L., Feng P.H., Lee K.Y. (2021). The Role of EREG/EGFR Pathway in Tumor Progression. Int J Mol Sci.

[49] Zhang Z., Shi R., Xu S. (2021). Identification of small proline-rich protein 1B (SPRR1B) as a prognostically predictive biomarker for lung adenocarcinoma by integrative bioinformatic analysis. Thorac Cancer.

[50] Jinesh G.G., Flores E.R., Brohl A.S. (2018). Chromosome 19 miRNA cluster and CEBPB expression specifically mark and potentially drive triple negative breast cancers. PLoS One.

[51] Kwong S.C., Abd J.A., Rhodes A., Taib N.A., Chung I. (2020). Fatty acid binding protein 7 mediates linoleic acid-induced cell death in triple negative breast cancer cells by modulating 13-HODE. Biochimie.

[52] Oh J.H., Kim C.Y., Jeong D.S. (2024). The homeoprotein HOXB2 limits triple-negative breast carcinogenesis via extracellular matrix remodeling. Int J Biol Sci.

[53] Bianchini G., Balko J.M., Mayer I.A., Sanders M.E., Gianni L. (2016). Triple-negative breast cancer: challenges and opportunities of a heterogeneous disease. Nat Rev Clin Oncol.

[54] Dekker E., Tanis P.J., Vleugels J., Kasi P.M., Wallace M.B. (2019). Colorectal cancer. Lancet.

[55] Medema J.P. (2013). Cancer stem cells: the challenges ahead. Nat Cell Biol.

[56] Nassar D., Blanpain C. (2016). Cancer stem cells: basic concepts and therapeutic implications. Annu Rev Pathol.

[57] Saha S., Mukherjee C., Basak D. (2023). High expression of mesothelin in plasma and tissue is associated with poor prognosis and promotes invasion and metastasis in gastric cancer. *Adv Cancer Biol* - Metastas--.

[58] Chen J., Chen J., Sun B., Wu J., Du C. (2020). ONECUT2 Accelerates Tumor Proliferation Through Activating ROCK1 Expression in Gastric Cancer. Cancer Manag Res.

[59] Jiang L., Wang X., Ma F. (2022). PITX2C increases the stemness features of hepatocellular carcinoma cells by up-regulating key developmental factors in liver progenitor. J Exp Clin Cancer Res.

[60] Katoh M., Katoh M. (2004). Human FOX gene family (Review). Int J Oncol.

[61] DeGregori J., Johnson D.G. (2006). Distinct and Overlapping Roles for E2F Family Members in Transcription, Proliferation and Apoptosis. Curr Mol Med.

[62] Ren B., Cam H., Takahashi Y. (2002). E2F integrates cell cycle progression with DNA repair, replication, and G(2)/M checkpoints. Genes Dev.

[63] Clarke M.F. (2019). Clinical and therapeutic implications of cancer stem cells. Reply *N Engl J Med*.

[64] Reya T., Morrison S.J., Clarke M.F., Weissman I.L. (2001). Stem cells, cancer, and cancer stem cells. Nature.

[65] Saygin C., Matei D., Majeti R., Reizes O., Lathia J.D. (2019). Targeting Cancer Stemness in the Clinic: From Hype to Hope. Cell Stem Cell.

[66] Yi X., Luo L., Zhu Y. (2022). SPP1 facilitates cell migration and invasion by targeting COL11A1 in lung adenocarcinoma. Cancer Cell Int.

[67] Tang H., Chen J., Han X., Feng Y., Wang F. (2021). Upregulation of SPP1 is a marker for poor lung cancer prognosis and contributes to cancer progression and cisplatin resistance. Front Cell Dev Biol.

[68] Luo A., Yin Y., Li X. (2015). The clinical significance of FSCN1 in non-small cell lung cancer. Biomed Pharm.

[69] Shi Y., Xu Y., Xu Z. (2022). TKI resistant-based prognostic immune related gene signature in LUAD, in which FSCN1 contributes to tumor progression. Cancer Lett.

[70] Gu C., Huang Z., Chen X. (2020). TEAD4 promotes tumor development in patients with lung adenocarcinoma via ERK signaling pathway. Biochim Biophys Acta Mol Basis Dis.

[71] Zhong P., Shu R., Wu H. (2021). Low KRT15 expression is associated with poor prognosis in patients with breast invasive carcinoma. Exp Ther Med.

[72] Yang B., Li M., Tang W. (2018). Dynamic network biomarker indicates pulmonary metastasis at the tipping point of hepatocellular carcinoma. Nat Commun.

[73] Wu Y., Liu H., Gong Y., Zhang B., Chen W. (2021). ANKRD22 enhances breast cancer cell malignancy by activating the Wnt/beta-catenin pathway via modulating NuSAP1 expression. Bosn J Basic Med Sci.

[74] Chen M.S., Lo Y.H., Chen X. (2019). Growth factor-independent 1 is a tumor suppressor gene in colorectal cancer. Mol Cancer Res.

[75] Wu S., Zhu D., Feng H. (2023). Comprehensive analysis of HOXC8 associated with tumor microenvironment characteristics in colorectal cancer. Heliyon.

[76] Pan Y., Zheng Y., Yang J. (2022). A new biomarker for the early diagnosis of gastric cancer: gastric juice- and serum-derived SNCG. Future Oncol.

[77] Shen C.T., Qiu Z.L., Song H.J., Wei W.J., Luo Q.Y. (2016). miRNA-106a directly targeting RARB associates with the expression of Na(+)/I(-) symporter in thyroid cancer by regulating MAPK signaling pathway. J Exp Clin Cancer Res.

[78] Moon S.J., Choi H.J., Kye Y.H. (2023). CTTN Overexpression Confers Cancer Stem Cell-like Properties and Trastuzumab Resistance via DKK-1/WNT Signaling in HER2 Positive Breast Cancer. Cancers (Basel).

[79] Jin B., Gong Z., Yang N. (2016). Downregulation of betaine homocysteine methyltransferase (BHMT) in hepatocellular carcinoma associates with poor prognosis. Tumour Biol.

[80] Liu H.Q., Sun L.X., Yu L. (2023). HSP90, as a functional target antigen of a mAb 11C9, promotes stemness and tumor progression in hepatocellular carcinoma. Stem Cell Res Ther.

[81] Grant S., Easley C., Kirkpatrick P. (2007). Vorinostat. Nat Rev Drug Discov.

[82] Marks P., Rifkind R.A., Richon V.M. (2001). Histone deacetylases and cancer: causes and therapies. Nat Rev Cancer.

[83] Secrist J.P., Zhou X., Richon V.M. (2003). HDAC inhibitors for the treatment of cancer. Curr Opin Invest Drugs.

[84] George P., Bali P., Annavarapu S. (2005). Combination of the histone deacetylase inhibitor LBH589 and the hsp90 inhibitor 17-AAG is highly active against human CML-BC cells and AML cells with activating mutation of FLT-3. Blood.

[85] Haura E.B., Ricart A.D., Larson T.G. (2010). A phase II study of PD-0325901, an oral MEK inhibitor, in previously treated patients with advanced non-small cell lung cancer. Clin Cancer Res.

[86] Moser C., Schachtschneider P., Lang S.A. (2008). Inhibition of insulin-like growth factor-I receptor (IGF-IR) using NVP-AEW541, a small molecule kinase inhibitor, reduces orthotopic pancreatic cancer growth and angiogenesis. Eur J Cancer.

[87] Gnanasundram S.V., Pyndiah S., Daskalogianni C. (2017). PI3Kdelta activates E2F1 synthesis in response to mRNA translation stress. Nat Commun.

[88] Clara J.A., Monge C., Yang Y., Takebe N. (2020). Targeting signalling pathways and the immune microenvironment of cancer stem cells - a clinical update. Nat Rev Clin Oncol.

[89] Wu Y., Zhou B.P. (2010). TNF-alpha/NF-kappaB/Snail pathway in cancer cell migration and invasion. Br J Cancer.

[90] Hayden M.S., Ghosh S. (2014). Regulation of NF-kappaB by TNF family cytokines. Semin Immunol.

[91] Shostak K., Chariot A. (2015). EGFR and NF-kappaB: partners in cancer. Trends Mol Med.

[92] Mirzaei S., Saghari S., Bassiri F. (2022). NF-kappaB as a regulator of cancer metastasis and therapy response: A focus on epithelial-mesenchymal transition. J Cell Physiol.

[93] Johnstone R.W., Frew A.J., Smyth M.J. (2008). The TRAIL apoptotic pathway in cancer onset, progression and therapy. Nat Rev Cancer.

[94] Ma B., Fey M., Hottiger M.O. (2015). WNT/beta-catenin signaling inhibits CBP-mediated RelA acetylation and expression of proinflammatory NF-kappaB target genes. J Cell Sci.

[95] Duan Y., Liao A.P., Kuppireddi S. (2007). beta-Catenin activity negatively regulates bacteria-induced inflammation. Lab Invest.

[96] Cho H.H., Song J.S., Yu J.M. (2008). Differential effect of NF-kappaB activity on beta-catenin/Tcf pathway in various cancer cells. FEBS Lett.

[97] Chen Y., Tian T., Li Z. (2019). FSCN1 is an effective marker of poor prognosis and a potential therapeutic target in human tongue squamous cell carcinoma. Cell Death Dis.

[98] Ren X.R., Wang J., Osada T. (2015). Perhexiline promotes HER3 ablation through receptor internalization and inhibits tumor growth. Breast Cancer Res.

[99] Wang Y., Lu J.H., Wang F. (2020). Inhibition of fatty acid catabolism augments the efficacy of oxaliplatin-based chemotherapy in gastrointestinal cancers. Cancer Lett.

[100] Kant S., Kesarwani P., Guastella A.R. (2020). Perhexiline Demonstrates FYN-mediated Antitumor Activity in Glioblastoma. Mol Cancer Ther.

[101] Xu S., Catapang A., Braas D. (2018). A precision therapeutic strategy for hexokinase 1-null, hexokinase 2-positive cancers. Cancer Metab.

[102] Schnell S.A., Ambesi-Impiombato A., Sanchez-Martin M. (2015). Therapeutic targeting of HES1 transcriptional programs in T-ALL. Blood.

[103] Platzbecker U., Ward J.L., Deeg H.J. (2003). Chelerythrin activates caspase-8, downregulates FLIP long and short, and overcomes resistance to tumour necrosis factor-related apoptosis-inducing ligand in KG1a cells. Br J Haematol.

[104] He H., Zhuo R., Dai J. (2020). Chelerythrine induces apoptosis via ROS-mediated endoplasmic reticulum stress and STAT3 pathways in human renal cell carcinoma. J Cell Mol Med.

[105] Zhou J., Wang Y., Fu Y. (2024). Chelerythrine induces apoptosis and ferroptosis through Nrf2 in ovarian cancer cells. Cell Mol Biol (Noisy-Le-Gd).

[106] Rinne M., Matlik K., Ahonen T. (2020). Mitoxantrone, pixantrone and mitoxantrone (2-hydroxyethyl)piperazine are toll-like receptor 4 antagonists, inhibit NF-kappaB activation, and decrease TNF-alpha secretion in primary microglia. Eur J Pharm Sci.

