# Supplementary material for: Tumour heterogeneity and personalized treatment screening based on single-cell transcriptomics
Source: Comput Struct Biotechnol J. 2024 Dec 25;27:307–20. doi: 10.1016/j.csbj.2024.12.020 (PMC11773088; doi:10.1016/j.csbj.2024.12.020)
Supplement: Supplementary file 1 — Supplementary material [file mmc1.pdf]

Supplementary materials

Tumour heterogeneity and personalized treatment screening based on single-cell transcriptomics

Xinying Zhang [a, ^], Jiajie Xie [a, ^], Zixin Yang [a], Carisa Kwok Wai Yu [b, *], Yaohua Hu [c, *], Jing Qin [a, *]

[a] School of Pharmaceutical Sciences (Shenzhen), Shenzhen Campus of Sun Yat-sen University, Shenzhen, Guangdong 518107, China

[b] Department of Mathematics, Statistics and Insurance, The Hang Seng University of Hong Kong, Shatin, Hong Kong

[c] Shenzhen Key Laboratory of Advanced Machine Learning and Applications, College of Mathematics and Statistics, Shenzhen University, Shenzhen, Guangdong 518060, China

^Equal contribution. *Corresponding authors.

Correspondence: carisayu@hsu.edu.hk; mayhhu@szu.edu.cn; qinj29@mail.sysu.edu.cn

Table of contents

Figure/Table	Page number
Figure S1. UMAPs based on the single-cell transcriptomes from 5 cancer types, color-coded by patient source.	4
Figure S2. UMAPs based on the single-cell transcriptomes from 5 cancer types, color-coded by sample origin.	5
Figure S3. UMAPs based on the single-cell transcriptomes from 5 cancer types, color-coded by main cell type.	6
Figure S4. UMAPs based on the single-cell transcriptomes from each patient with lung adenocarcinoma (LUAD).	7
Figure S5. UMAPs based on the single-cell transcriptomes from each patient with breast cancer (BC).	8
Figure S6. UMAPs based on the single-cell transcriptomes from each patient with colorectal cancer (CRC).	9
Figure S7. UMAPs based on the single-cell transcriptomes from each patient with gastric cancer (GC).	10
Figure S8. UMAPs based on the single-cell transcriptomes from each patient with hepatocellular carcinoma (HCC).	11
Figure S9. Heatmap of chromosomal CNV status in each cell subtype from lung adenocarcinoma (LUAD).	12
Figure S10. Heatmap of chromosomal CNV status in each cell subtype from breast cancer (BC).	13
Figure S11. Heatmap of chromosomal CNV status in each cell subtype from colorectal cancer (CRC).	14
Figure S12. Heatmap of chromosomal CNV status in each cell subtype from gastric cancer (GC).	15
Figure S13. Heatmap of chromosomal CNV status in each cell subtype from hepatocellular carcinoma (HCC).	16
Figure S14. Biological differences among tumour cell subtypes from breast cancer (BC).	17
Figure S15. Biological differences among tumour cell subtypes from colorectal cancer (CRC).	18
Figure S16. Biological differences among tumour cell subtypes from gastric cancer (GC).	19
Figure S17. Biological differences among tumour cell subtypes from hepatocellular carcinoma (HCC).	20
Figure S18. Heatmap of GSVA pathway activity in each tumour cell subtype from lung adenocarcinoma (LUAD).	21
Figure S19. Heatmap of GSVA pathway activity in each tumour cell subtype from breast cancer (BC).	22
Figure S20. Heatmap of GSVA pathway activity in each tumour cell subtype from colorectal cancer (CRC).	23

Figure S21. Heatmap of GSVA pathway activity in each tumour cell subtype from gastric cancer (GC).	24
Figure S22. Heatmap of GSVA pathway activity in each tumour cell subtype from hepatocellular carcinoma (HCC).	25
Figure S23. Specific biomarkers of tumour cell subtypes from breast cancer (BC).	26
Figure S24. Specific biomarkers of tumour cell subtypes from colorectal cancer (CRC).	28
Figure S25. Specific biomarkers of tumour cell subtypes from gastric cancer (GC).	30
Figure S26. Specific biomarkers of tumour cell subtypes from hepatocellular carcinoma (HCC).	32
Figure S27. Specific biomarkers of tumour cell subtypes from lung adenocarcinoma (LUAD) after validation of independent datasets.	34
Figure S28. Specific biomarkers of tumour cell subtypes from breast cancer (BC) after validation of independent datasets.	35
Figure S29. Specific biomarkers of tumour cell subtypes from colorectal cancer (CRC) after validation of independent datasets.	36
Figure S30. Specific biomarkers of tumour cell subtypes from gastric cancer (GC) after validation of independent datasets.	37
Figure S31. Specific biomarkers of tumour cell subtypes from hepatocellular carcinoma (HCC) after validation of independent datasets.	38
Table S1. Source of datasets.	In a separate excel file
Table S2. The marker genes for cell type annotation.	In a separate excel file
Table S3. The top 10 DEGs of each subcluster.	In a separate excel file
Table S4. Reprehensive DEGs located in CNV regions.	In a separate excel file
Table S5. The top 10 key TFs of each subcluster.	In a separate excel file
Table S6. Cancer genes regulated by key TFs.	In a separate excel file
Table S7. The specific biomarkers identified for each subcluster.	In a separate excel file
Table S8. The specific drugs matched with each subcluster.	In a separate excel file

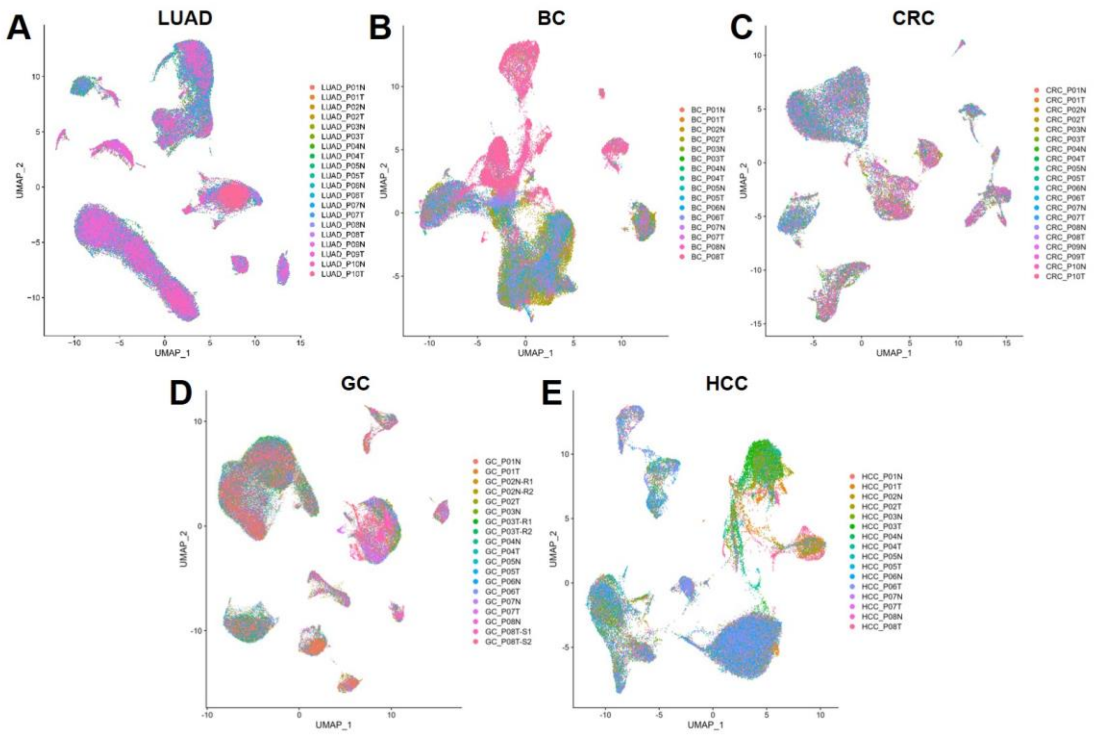

Figure S1. UMAPs based on the single-cell transcriptomes from 5 cancer types (A) lung adenocarcinoma (LUAD), (B) breast cancer (BC), (C) colorectal cancer (CRC), (D) gastric cancer (GC), (E) hepatocellular carcinoma (HCC), color-coded by patient source. Cells were colored by patient samples. Each sample ID starts from cancer type: LUAD, BC, CRC, GC or HCC; followed by sample number P01-P10; after that “T” means tumour tissue sample, “N” means adjacent normal tissue sample; "R1" and "R2" as well as "S1" and "S2" represent sampling from different tissue sites. After the quality control, batch effect correction, and dimensionality reduction, single-cell transcriptome data of each cancer type from different sample sources were well integrated.

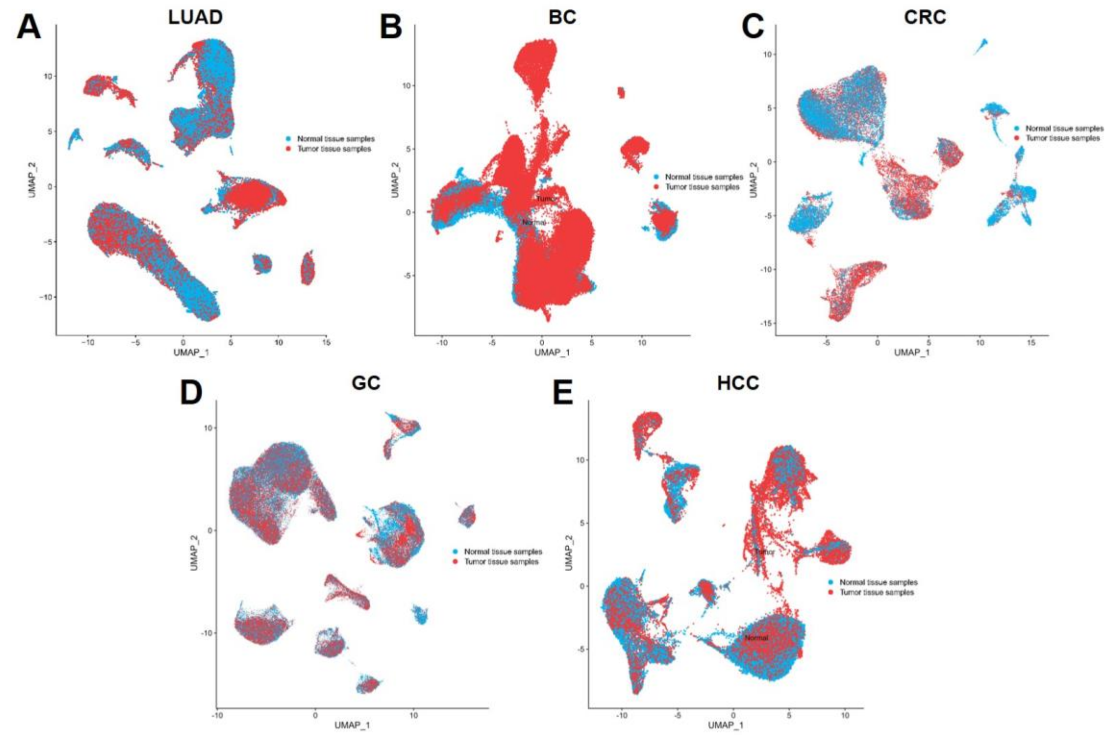

Figure S2. UMAPs based on the single-cell transcriptomes from 5 cancer types (A) lung adenocarcinoma (LUAD), (B) breast cancer (BC), (C) colorectal cancer (CRC), (D) gastric cancer (GC), (E) hepatocellular carcinoma (HCC), color-coded by sample origin (normal tissue samples and tumour tissue samples).

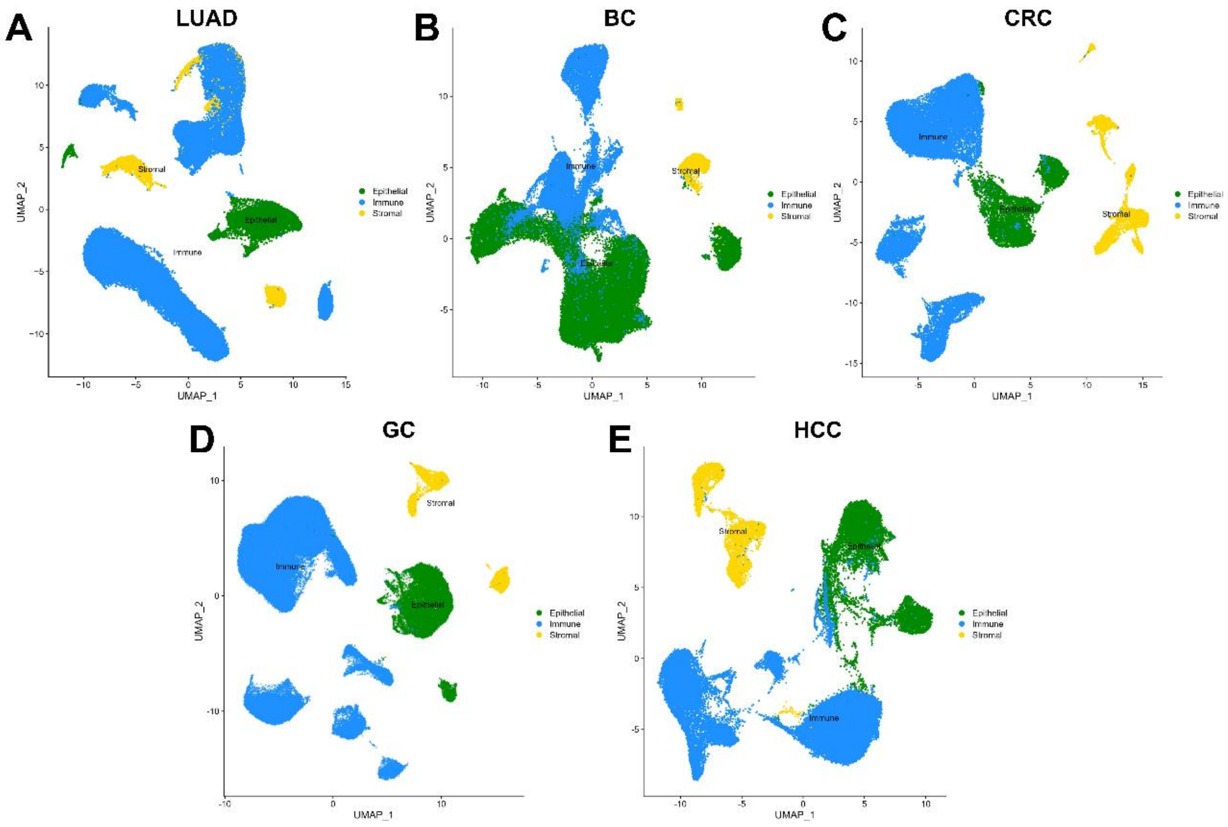

Figure S3. UMAPs based on the single-cell transcriptomes from 5 cancer types (A) lung adenocarcinoma (LUAD), (B) breast cancer (BC), (C) colorectal cancer (CRC), (D) gastric cancer (GC), (E) hepatocellular carcinoma (HCC), color-coded by main cell type. Stromal cells, epithelial cells, and immune cells were annotated according to their markers: EPCAM, SFN, KRT19 (epithelial cells), PTPRC, CD3E, CD79A (immune cells), and PECAM1, CD34, VWF, ACTA2, FAP, THY1 (stromal cells).

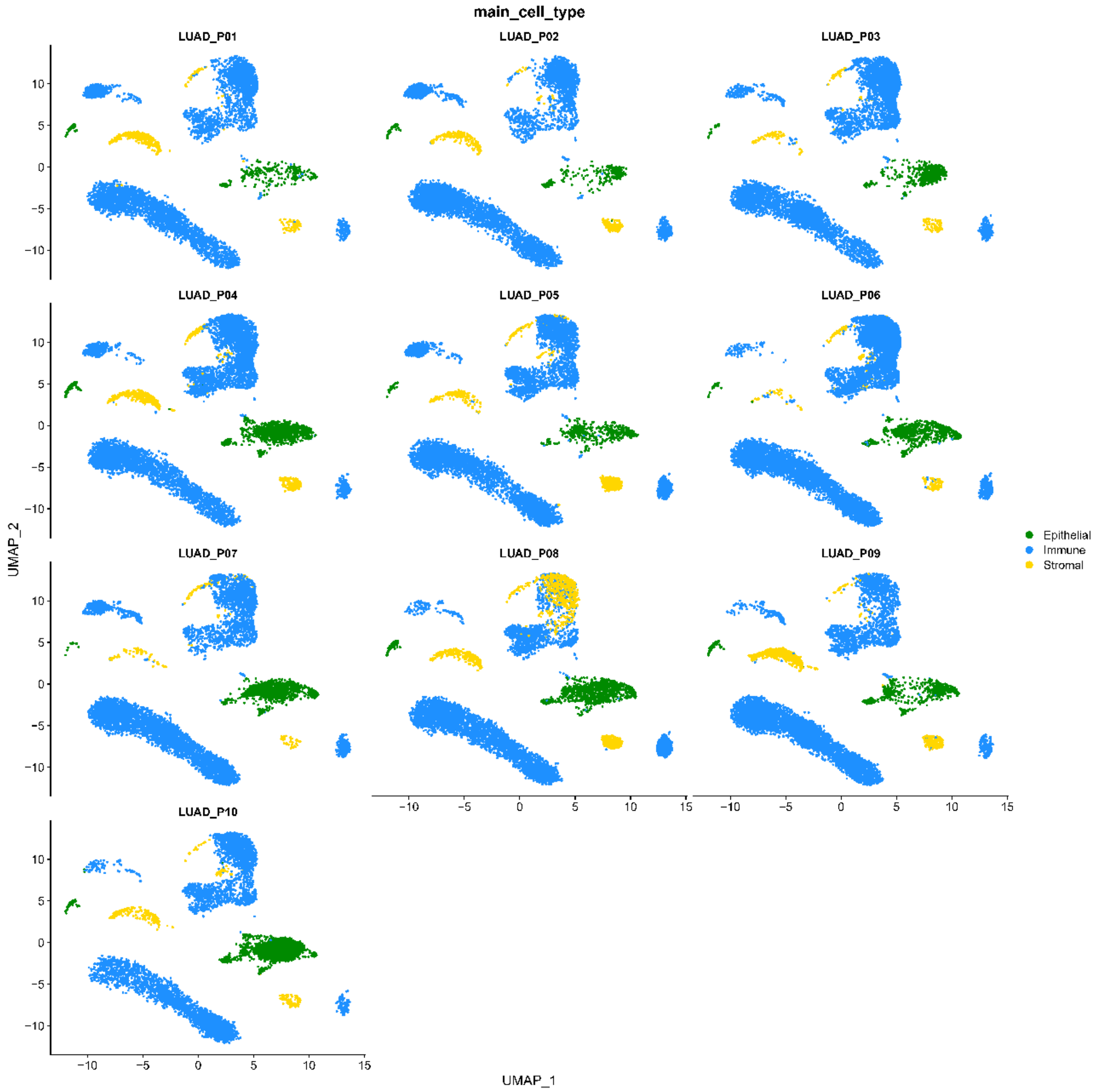

Figure S4. UMAPs based on the single-cell transcriptomes from each patient with lung adenocarcinoma (LUAD). All cells from each patient were annotated as three cell types: stromal cells, epithelial cells, and immune cells. Cells of the same cell type from different patients almost clustered together, indicating that the batch effects have been effectively eliminated.

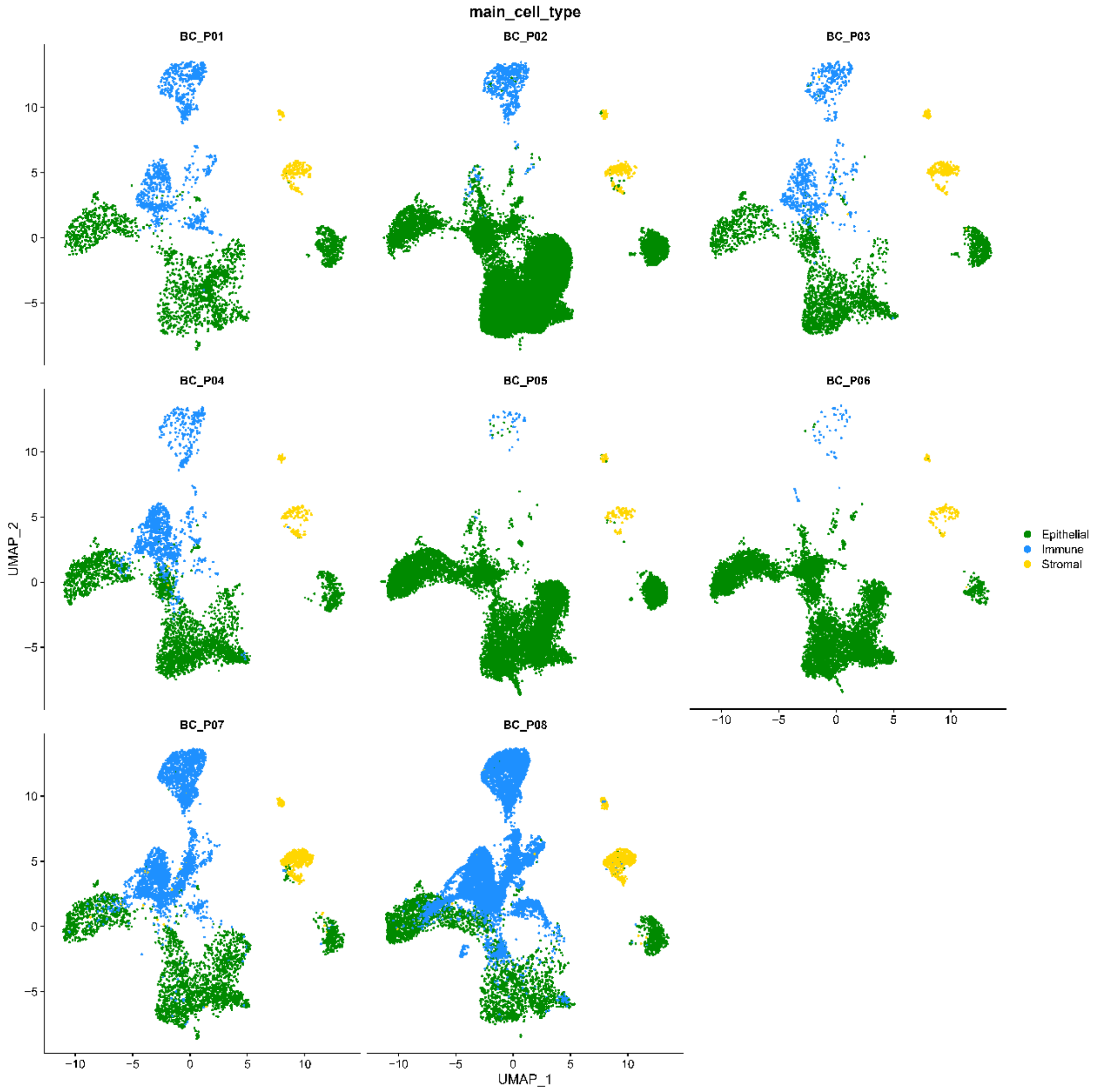

Figure S5. UMAPs based on the single-cell transcriptomes from each patient with breast cancer (BC). All cells from each patient were annotated as three cell types: stromal cells, epithelial cells, and immune cells. Cells of the same cell type from different patients almost clustered together, indicating that the batch effects have been effectively eliminated.

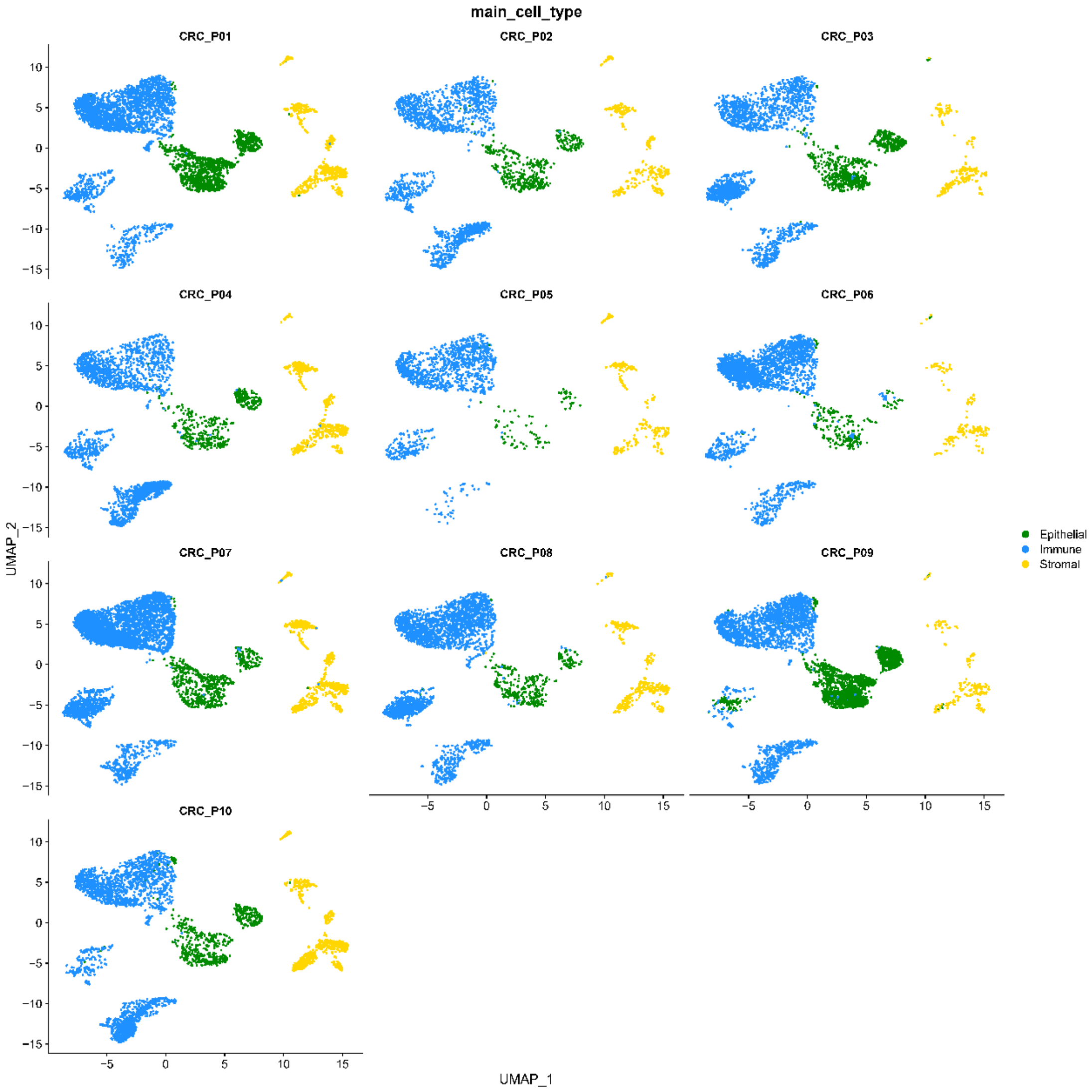

Figure S6. UMAPs based on the single-cell transcriptomes from each patient with colorectal cancer (CRC). All cells from each patient were annotated as three cell types: stromal cells, epithelial cells, and immune cells. Cells of the same cell type from different patients almost clustered together, indicating that the batch effects have been effectively eliminated.

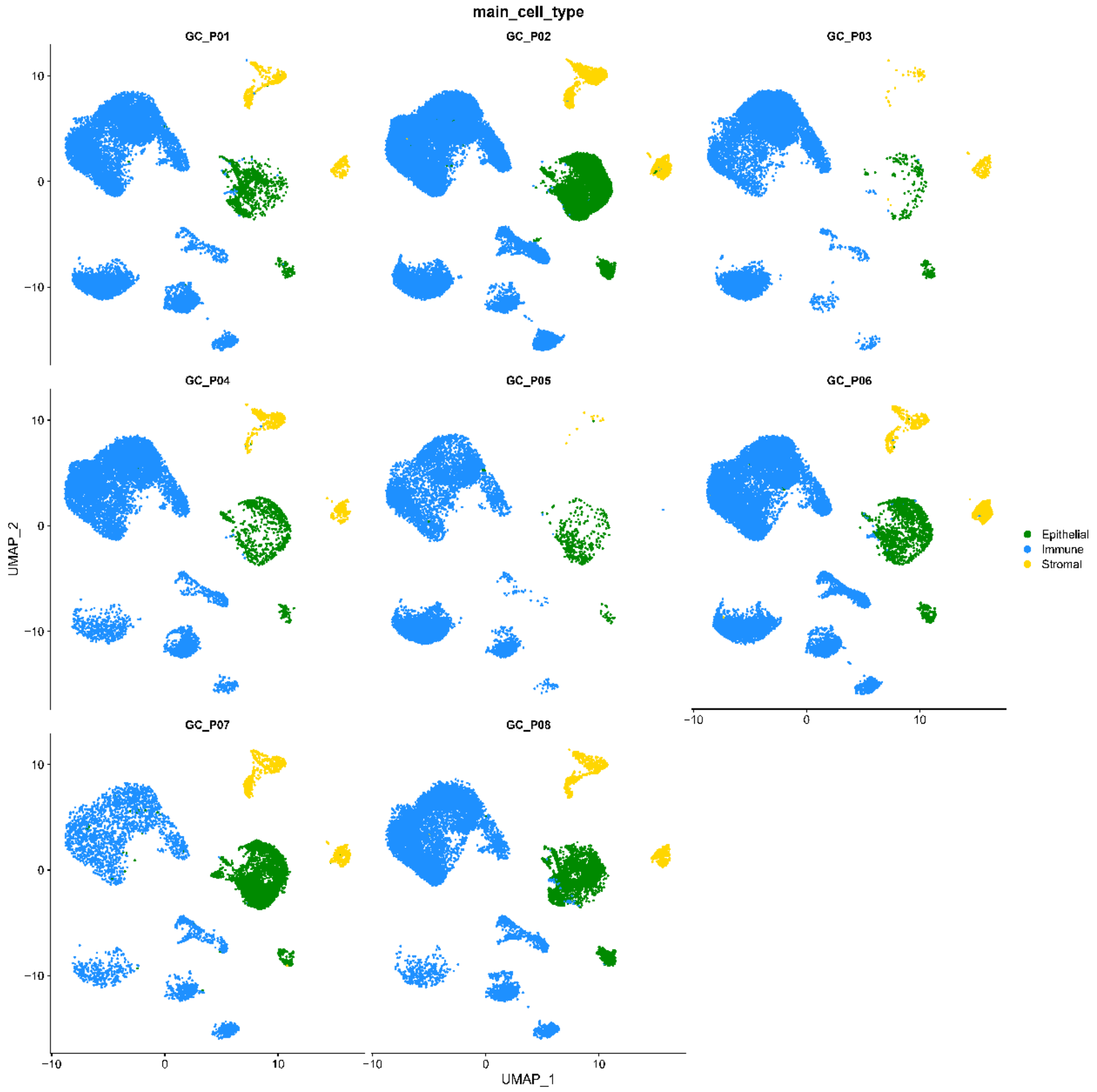

Figure S7. UMAPs based on the single-cell transcriptomes from each patient with gastric cancer (GC). All cells from each patient were annotated as three cell types: stromal cells, epithelial cells, and immune cells. Cells of the same cell type from different patients almost clustered together, indicating that the batch effects have been effectively eliminated.

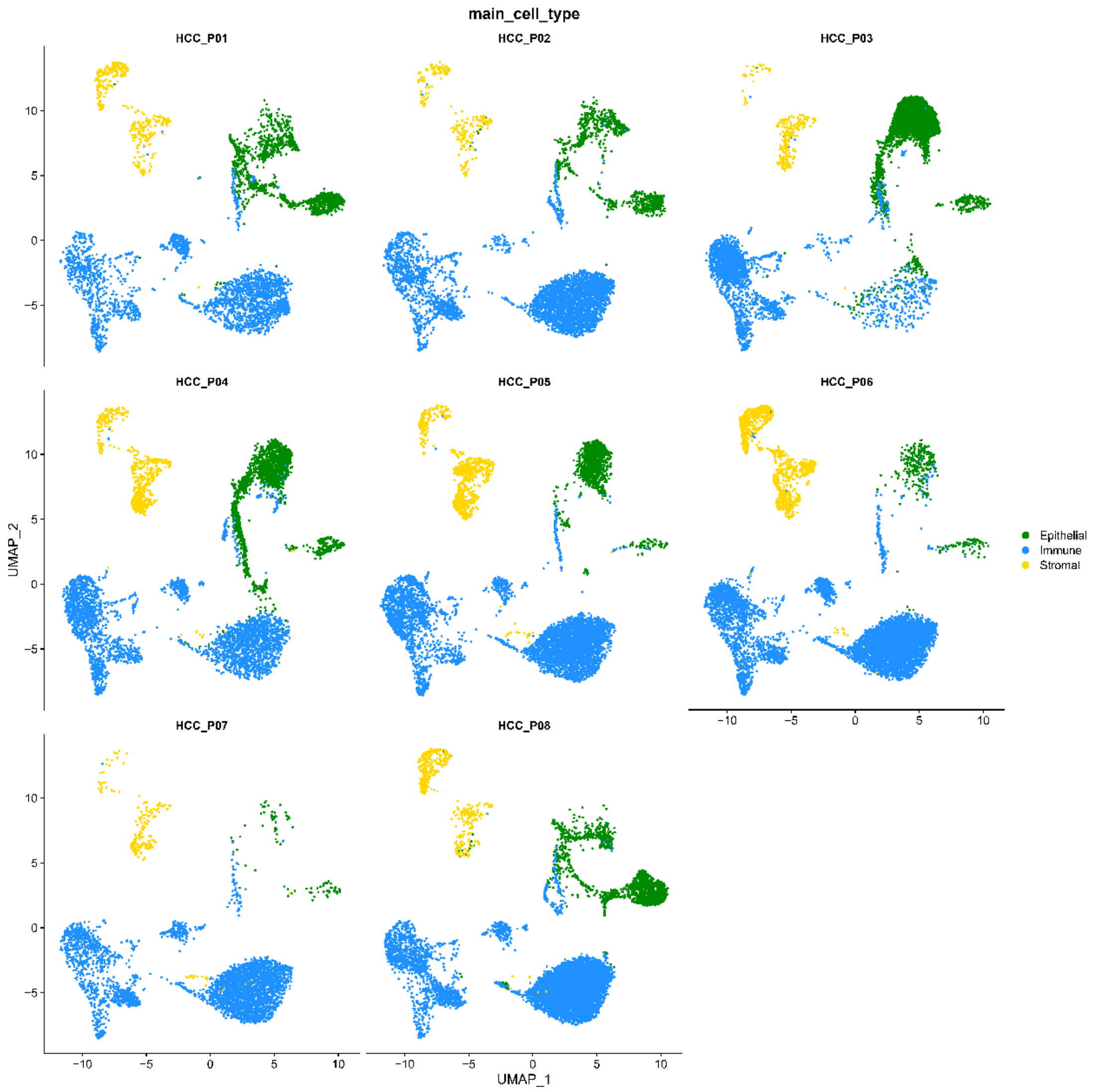

Figure S8. UMAPs based on the single-cell transcriptomes from each patient with hepatocellular carcinoma (HCC). All cells from each patient were annotated as three cell types: stromal cells, epithelial cells, and immune cells. Cells of the same cell type from different patients almost clustered together, indicating that the batch effects have been effectively eliminated.

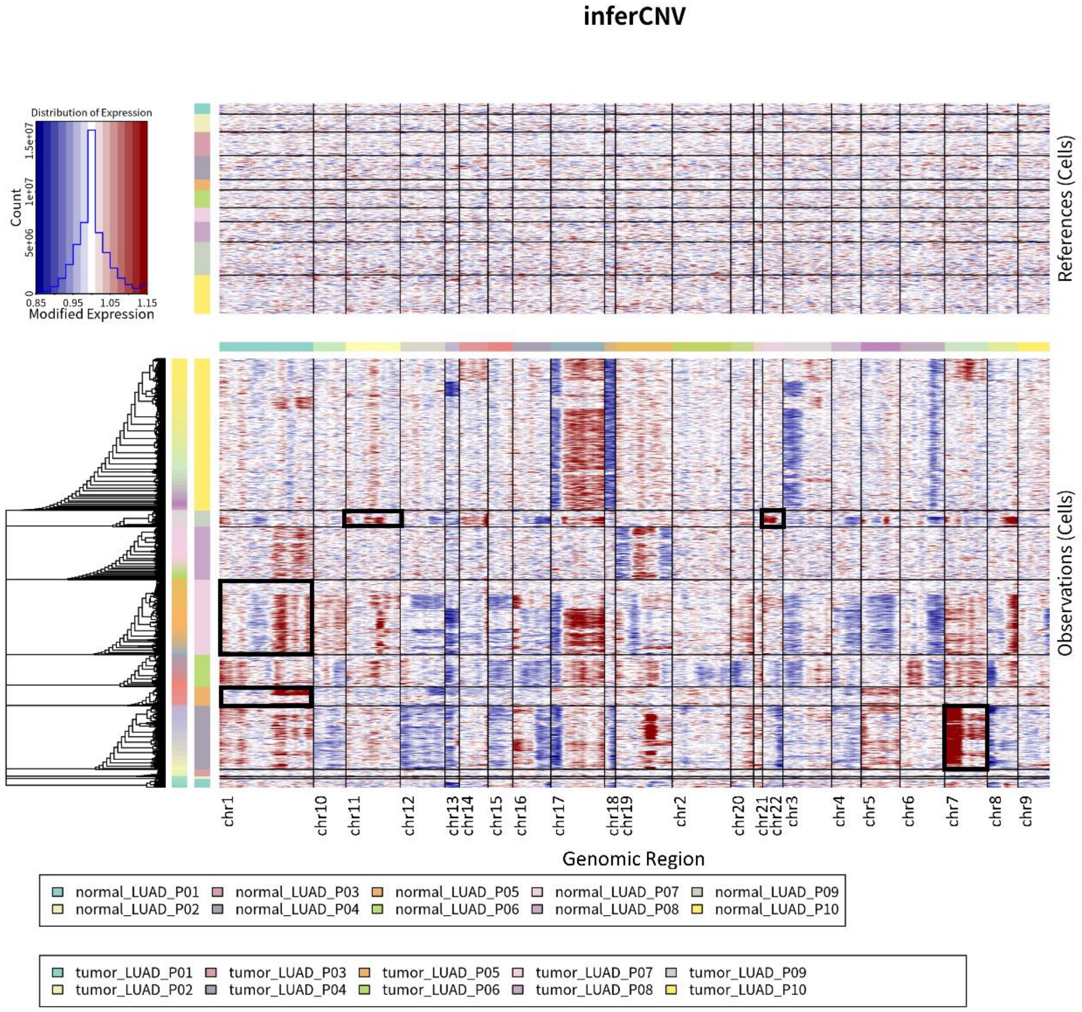

Figure S9. Heatmap of chromosomal CNV status in each cell subtype from lung adenocarcinoma (LUAD). tumour cells from patient LUAD_P05 exhibited substantial amplification on chromosome 1, while tumour cells from LUAD_P04 and LUAD_P07 showed notable amplifications on chromosomes 7 and 17, respectively. The black-framed regions are those mentioned in Table S4.

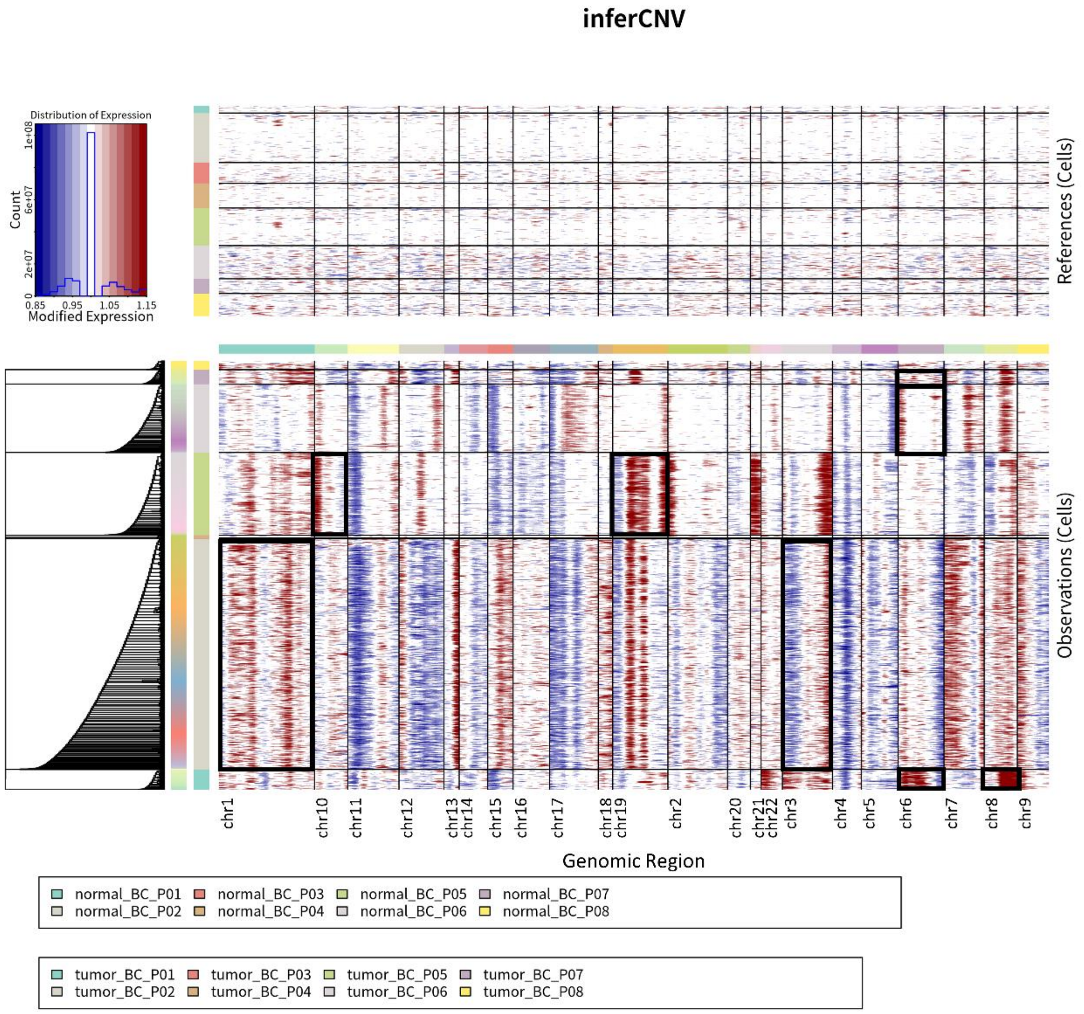

Figure S10. Heatmap of chromosomal CNV status in each cell subtype from breast cancer (BC). Abnormal copy number variations were detected on chromosomes 3, 6, 8, and 22 in tumour cells from BC_P01, and on chromosomes 2, 3, 19, and 21 in tumour cells from BC_P05. The black-framed regions are those mentioned in Table S4.

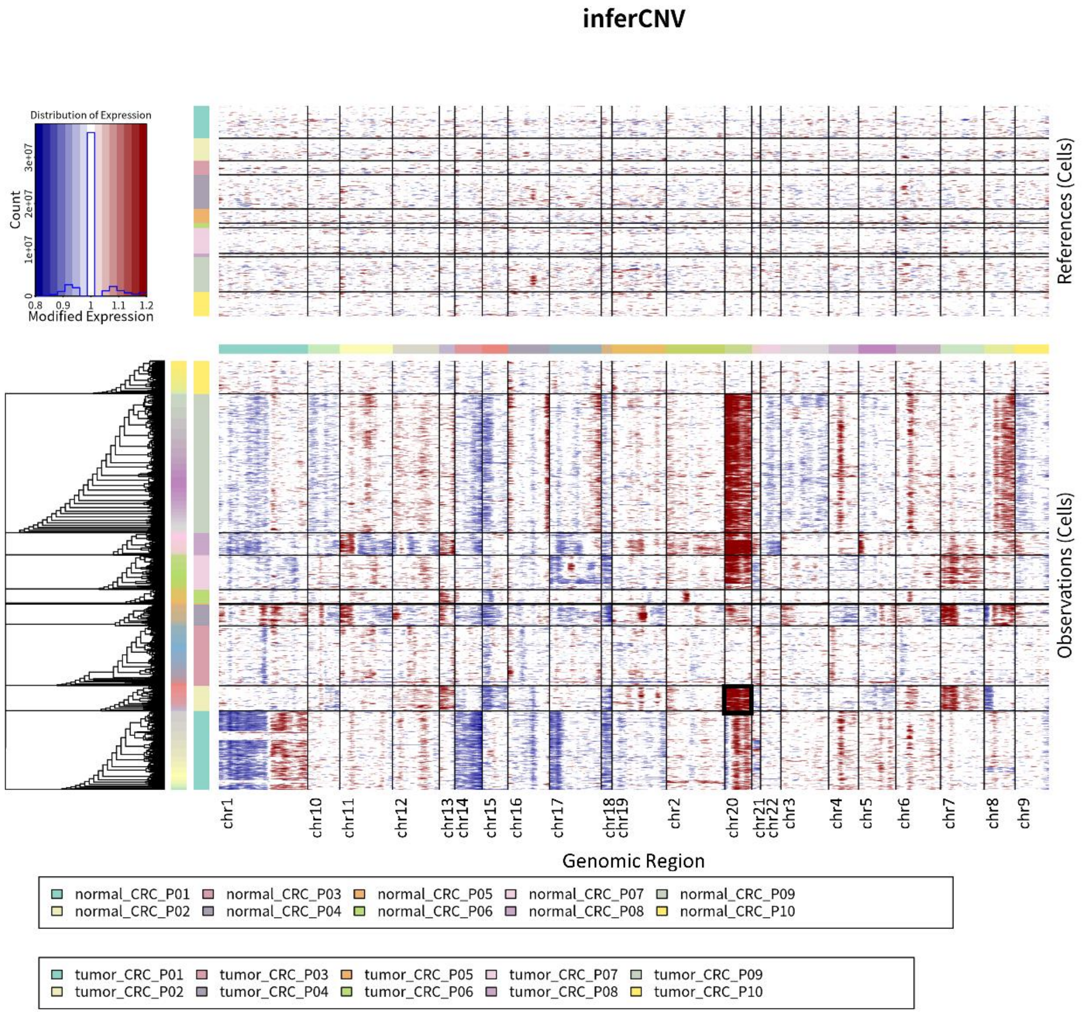

Figure S11. Heatmap of chromosomal CNV status in each cell subtype from colorectal cancer (CRC). tumour cells from CRC_P02 and CRC_P09 both exhibited significant abnormal amplifications on chromosome 20. The black-framed regions are those mentioned in Table S4.

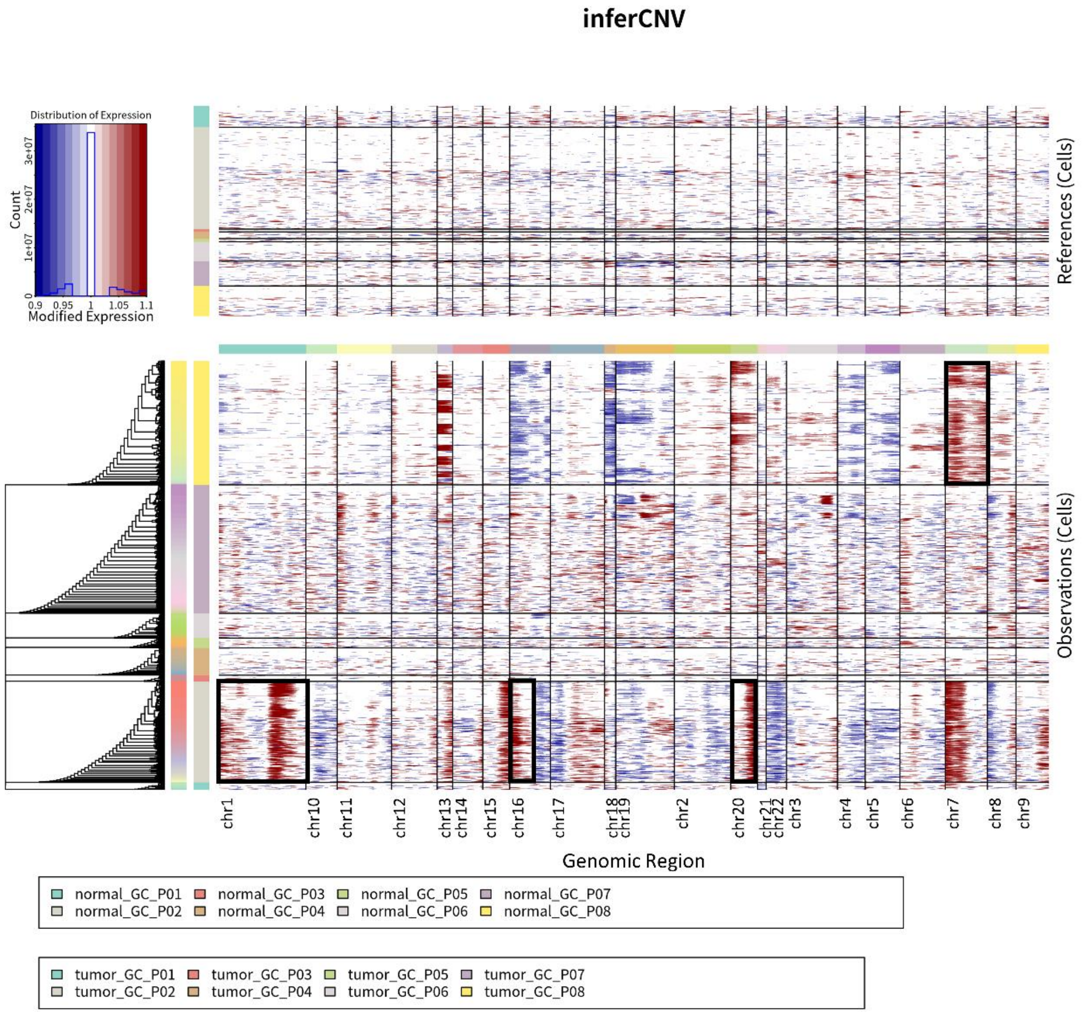

Figure S12. Heatmap of chromosomal CNV status in each cell subtype from gastric cancer (GC). Notable abnormal amplifications were detected on chromosomes 16 and 20 in tumour cells from GC_P02. The black-framed regions are those mentioned in Table S4.

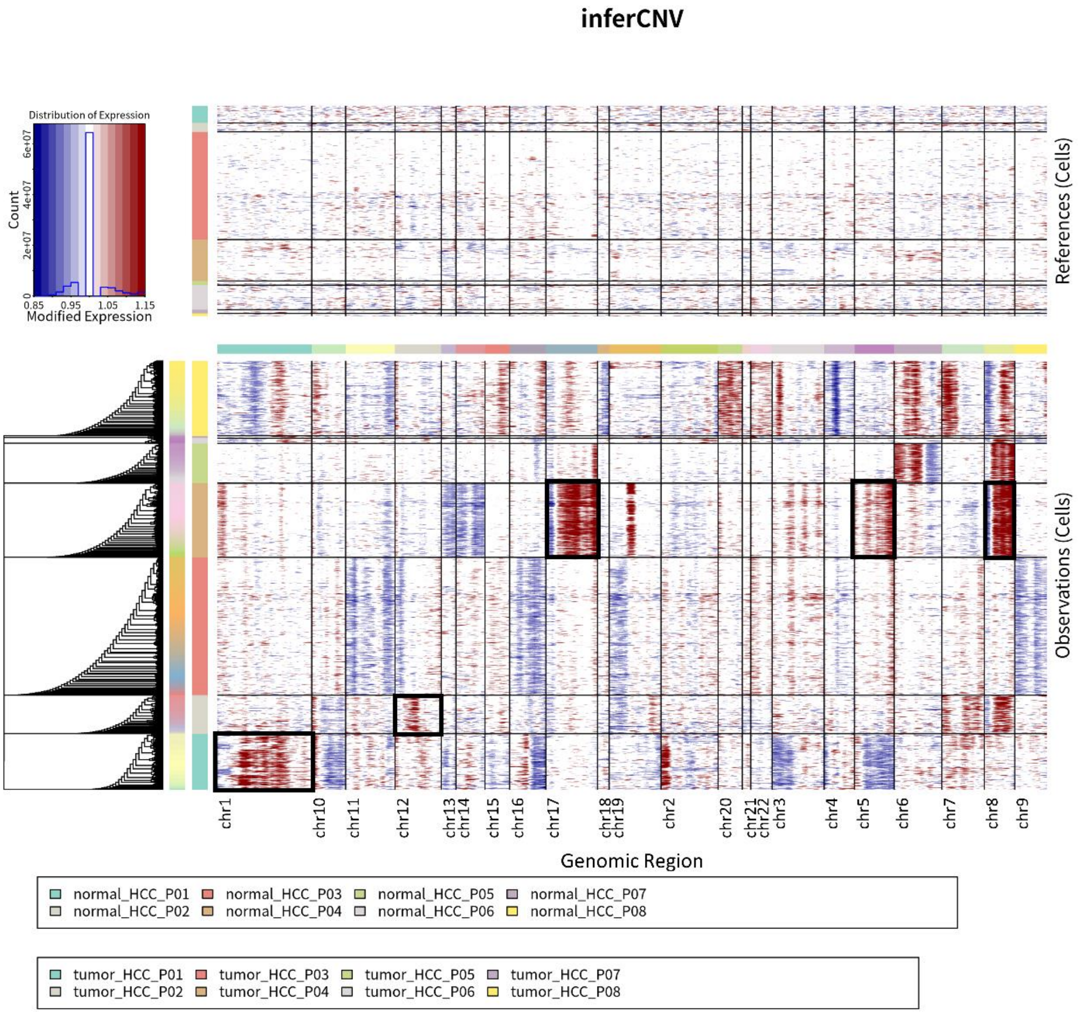

Figure S13. Heatmap of chromosomal CNV status in each cell subtype from hepatocellular carcinoma (HCC). Abnormal amplifications were observed on chromosome 8 in tumour cells from HCC_P04. The black-framed regions are those mentioned in Table S4.

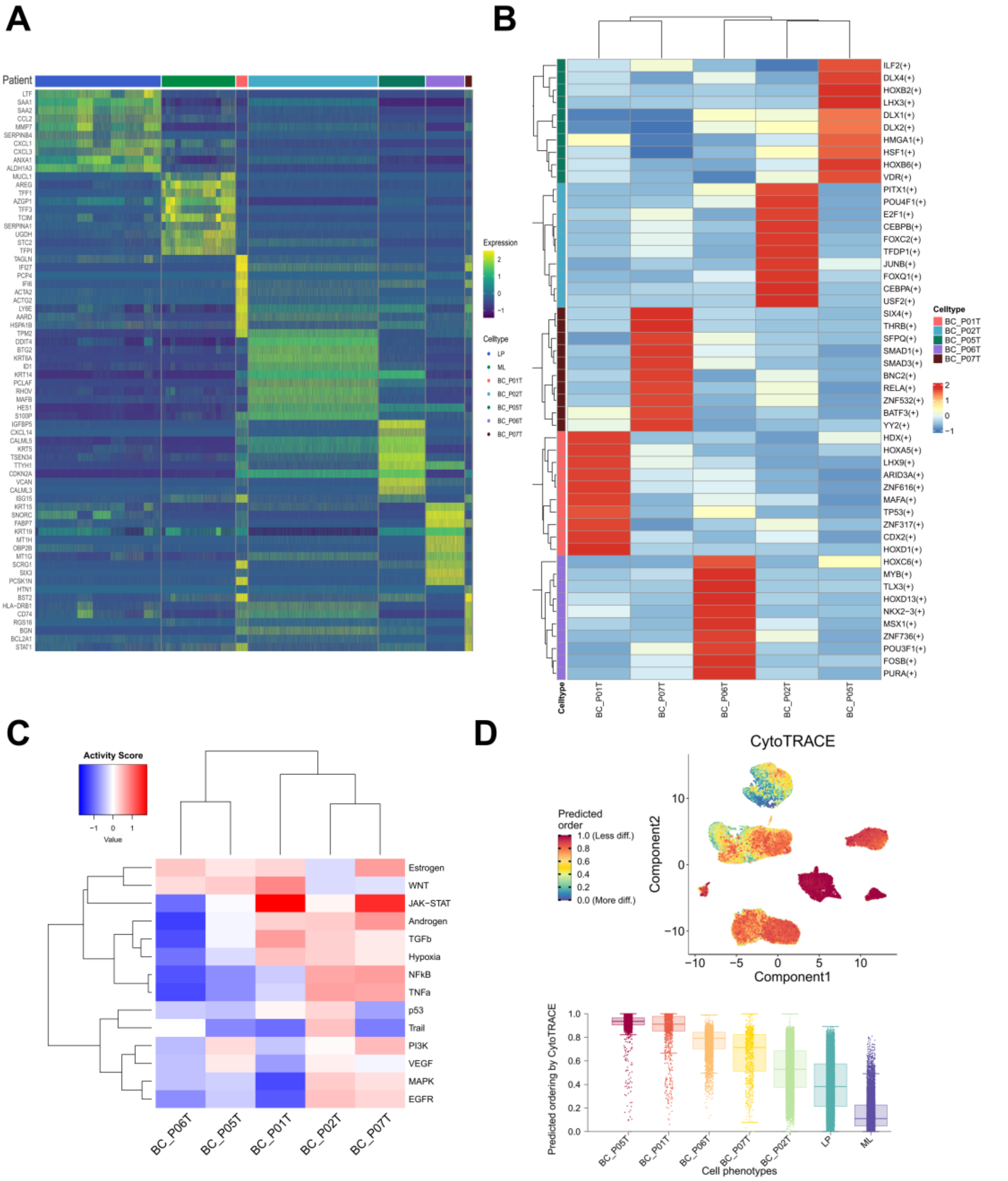

Figure S14. Biological differences among tumour cell subtypes from breast cancer (BC). Heatmaps of (A) the top 10 DEGs, (B) the top 10 key TFs, and (C) PROGENy pathway activity in each tumour cell subtype. (D) UMAP and box chart based on differentiation potential score. Noteworthy differences were observed in the activity levels of the JAK/STAT, TNF-α, NFκB and p53 signaling pathways across the diverse tumour cell subclusters. tumour cells exhibited a greater differentiation potential in comparison to luminal progenitor cells and mature luminal cells within the epithelial cell population. In particular, BC_P05T and BC_P01T demonstrated a higher differentiation potential.

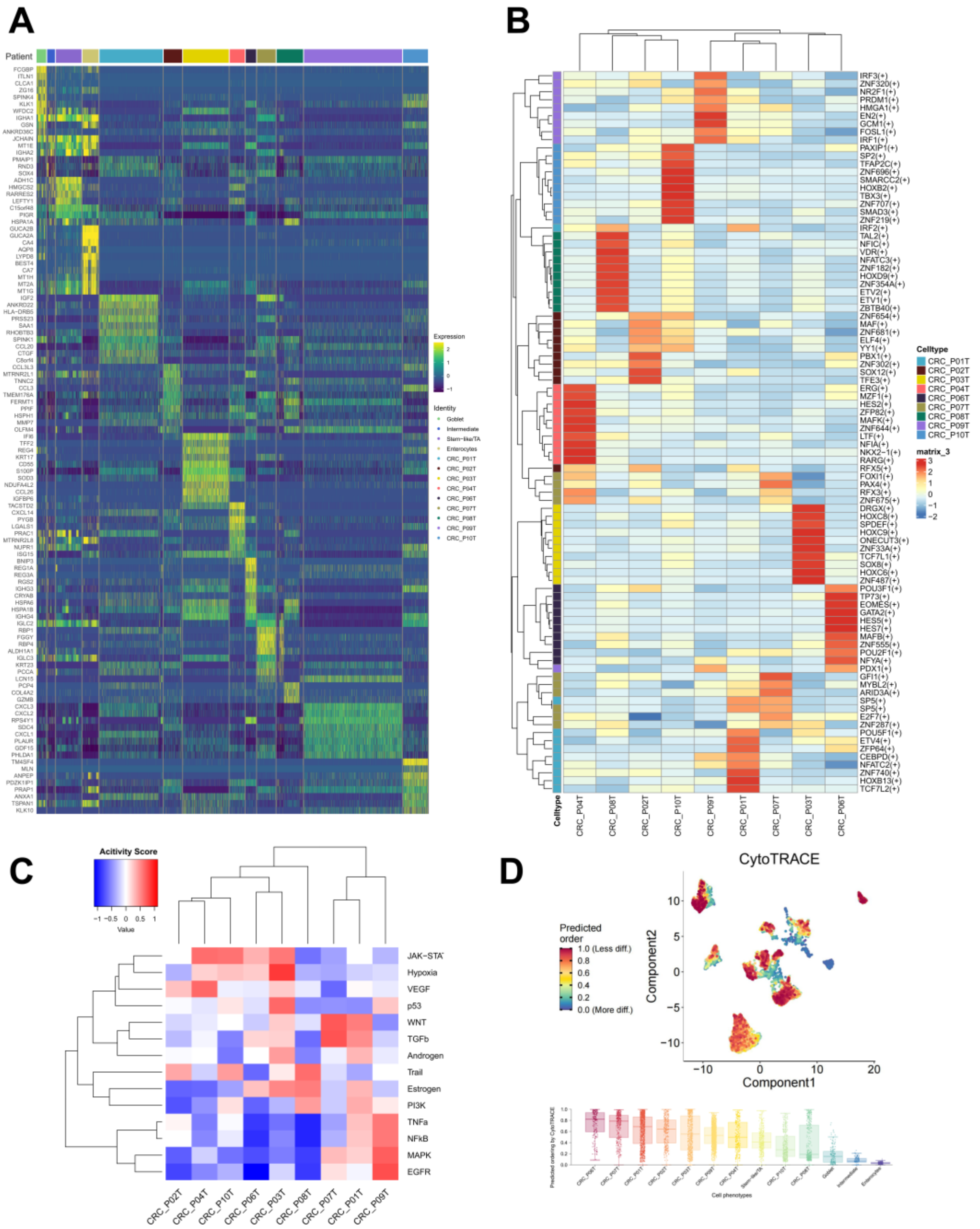

Figure S15. Biological differences among tumour cell subtypes from colorectal cancer (CRC). Heatmaps of (A) the top 10 DEGs, (B) the top 10 key TFs, and (C) PROGENy pathway activity in each tumour cell subtype. (D) UMAP and box chart based on differentiation potential score. Noteworthy differences were observed in the activity levels of the JAK/STAT, TNF-α, NFκB, MAPK and EGFR signaling pathways across the diverse tumour cell subclusters. tumour cells exhibited a greater differentiation potential in comparison to globet cells, intermediate and enterocytes within the epithelial cell population. In particular, CRC_P06T, CRC_P07T and CRC_P09T demonstrated a higher differentiation potential. CRC_P10T and CRC_P08T showed a lower differentiation potential.

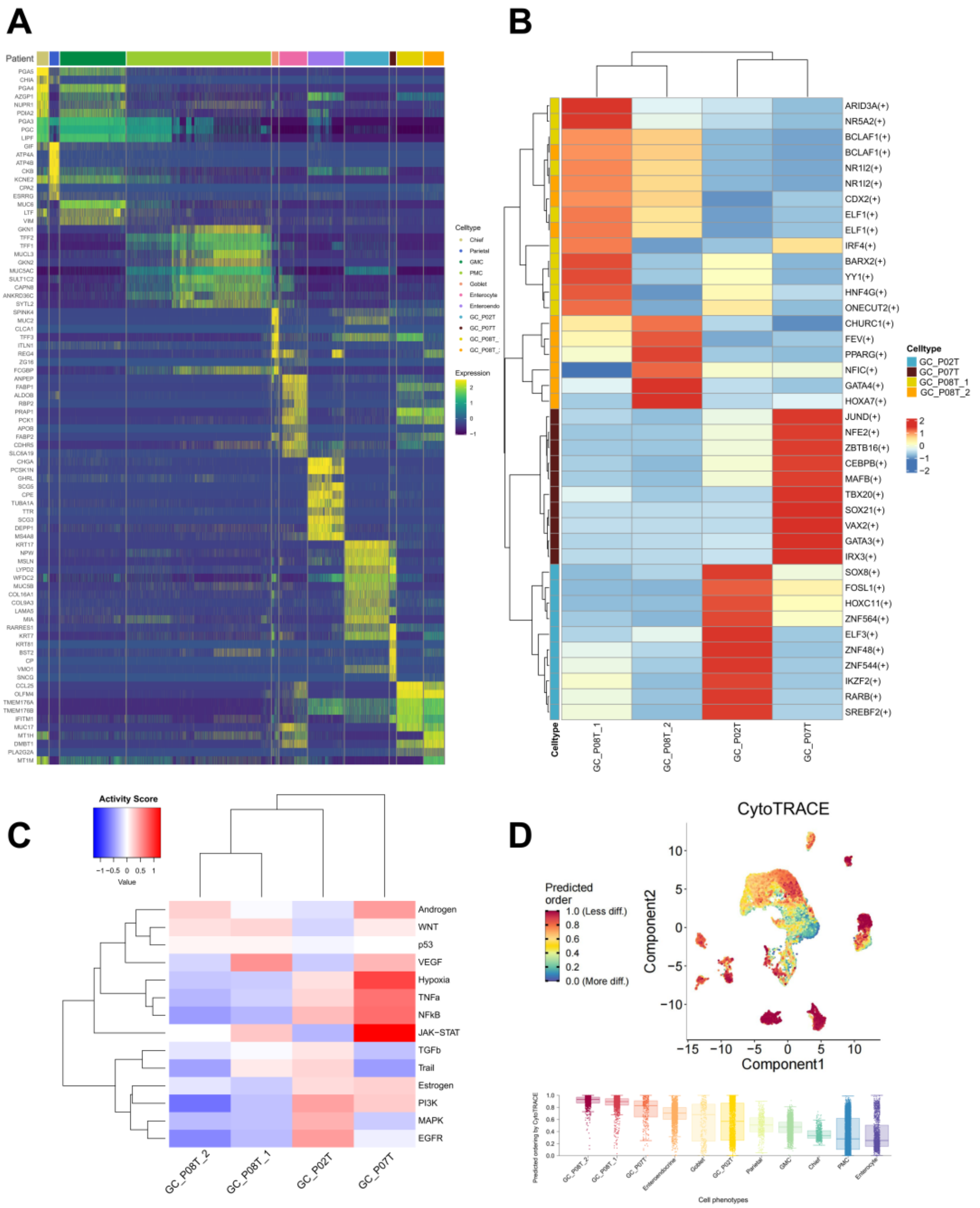

Figure S16. Biological differences among tumour cell subtypes from gastric cancer (GC). Heatmaps of (A) the top 10 DEGs, (B) the top 10 key TFs, and (C) PROGENy pathway activity in each tumour cell subtype. (D) UMAP and box chart based on differentiation potential score. Noteworthy differences were observed in the activity levels of the TNF-α, NFκB and JAK/STAT signaling pathways across the diverse tumour cell subclusters. tumour cells exhibited a greater differentiation potential in comparison to normal epithelial cells. In particular, GC_P08T_2 and GC_P08T_1 demonstrated a higher differentiation potential.

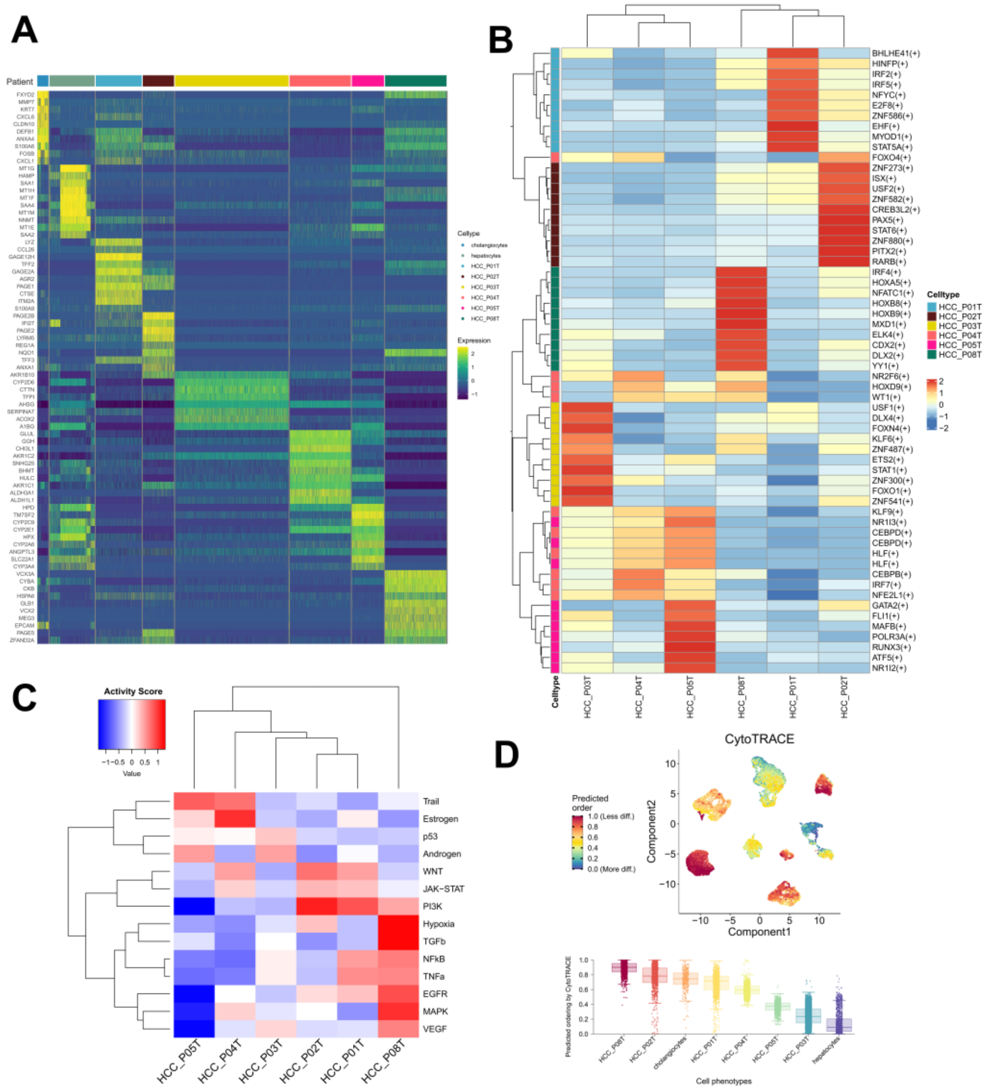

Figure S17. Biological differences among tumour cell subtypes from hepatocellular carcinoma (HCC). Heatmaps of (A) the top 10 DEGs, (B) the top 10 key TFs, and (C) PROGENy pathway activity in each tumour cell subtype. (D) UMAP and box chart based on differentiation potential score. Noteworthy differences were observed in the activity levels of the TNF-α, NFκB and EGFR signaling pathways across the diverse tumour cell subclusters. tumour cells exhibited a greater differentiation potential in comparison to normal epithelial cells. In particular, HCC_P08T and HCC_P02T demonstrated a higher differentiation potential.

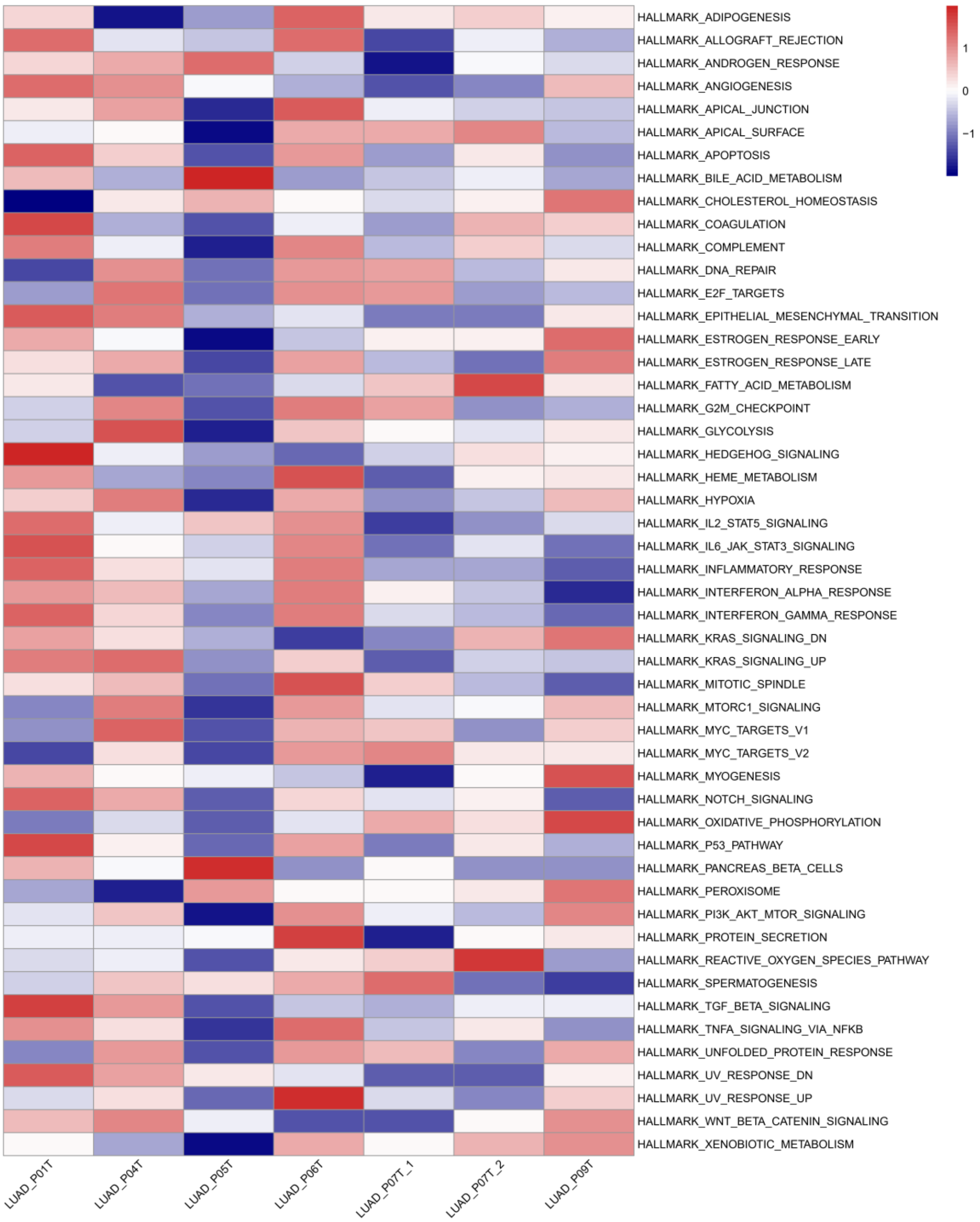

Figure S18. Heatmap of GSVA pathway activity in each tumour cell subtype from lung adenocarcinoma (LUAD). LUAD_P06T with NFκB-active property (active TNFA_Signaling_via_NFκB) showed higher activities of gene sets related to cell proliferation, such as Mitotic spindle and G2M checkpoint, and higher activities of gene sets related to inflammation, such as inflammation response and IL2/6 signaling.

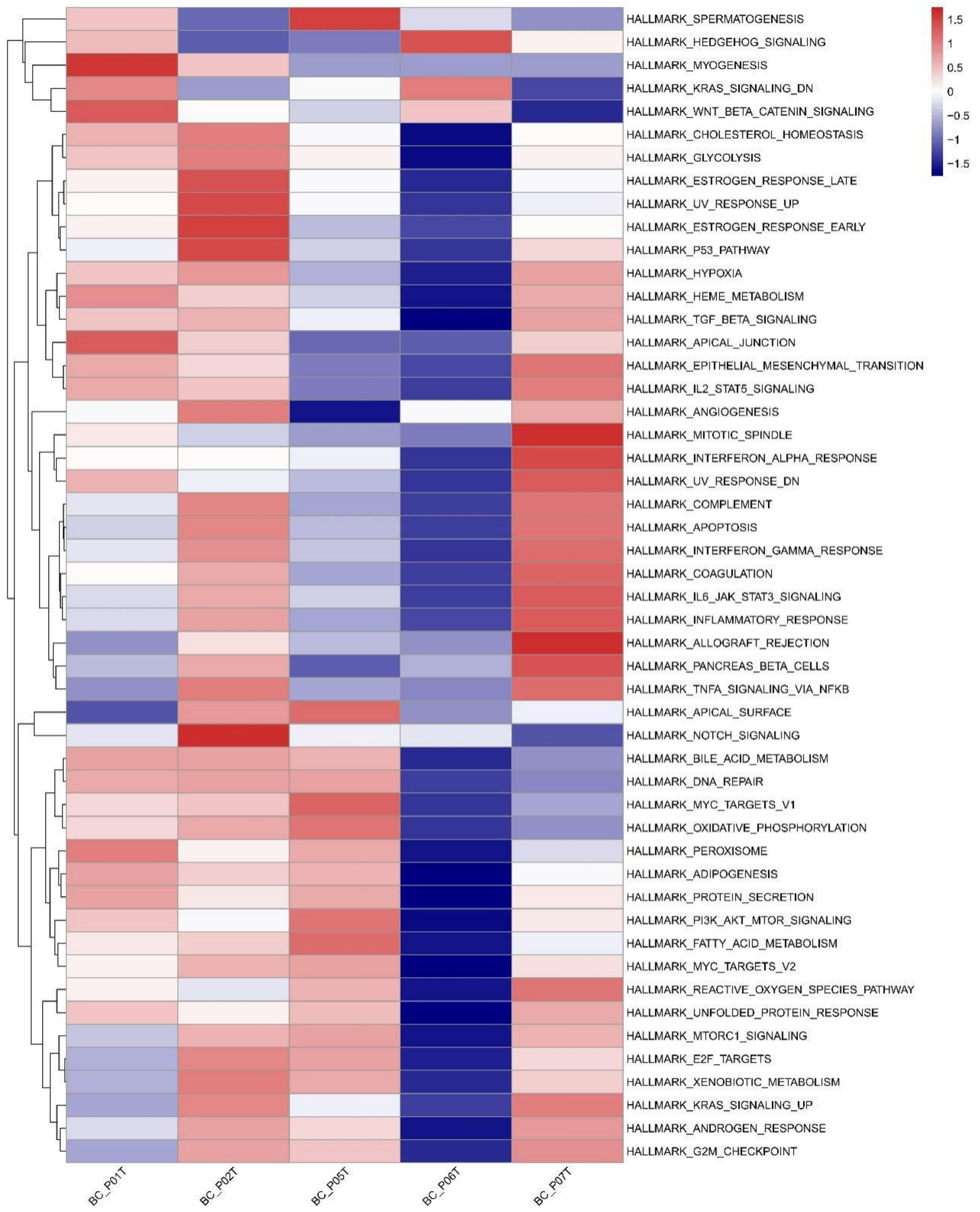

Figure S19. Heatmap of GSVA pathway activity in each tumour cell subtype from breast cancer (BC). BC_P02T and BC_P07T with NFκB-active property (active TNFA_Signaling_via_NFκB) showed higher activities of gene sets related to cell proliferation, such as Mitotic spindle and G2M checkpoint, and higher activities of gene sets related to inflammation, such as inflammation response and IL2/6 signaling.

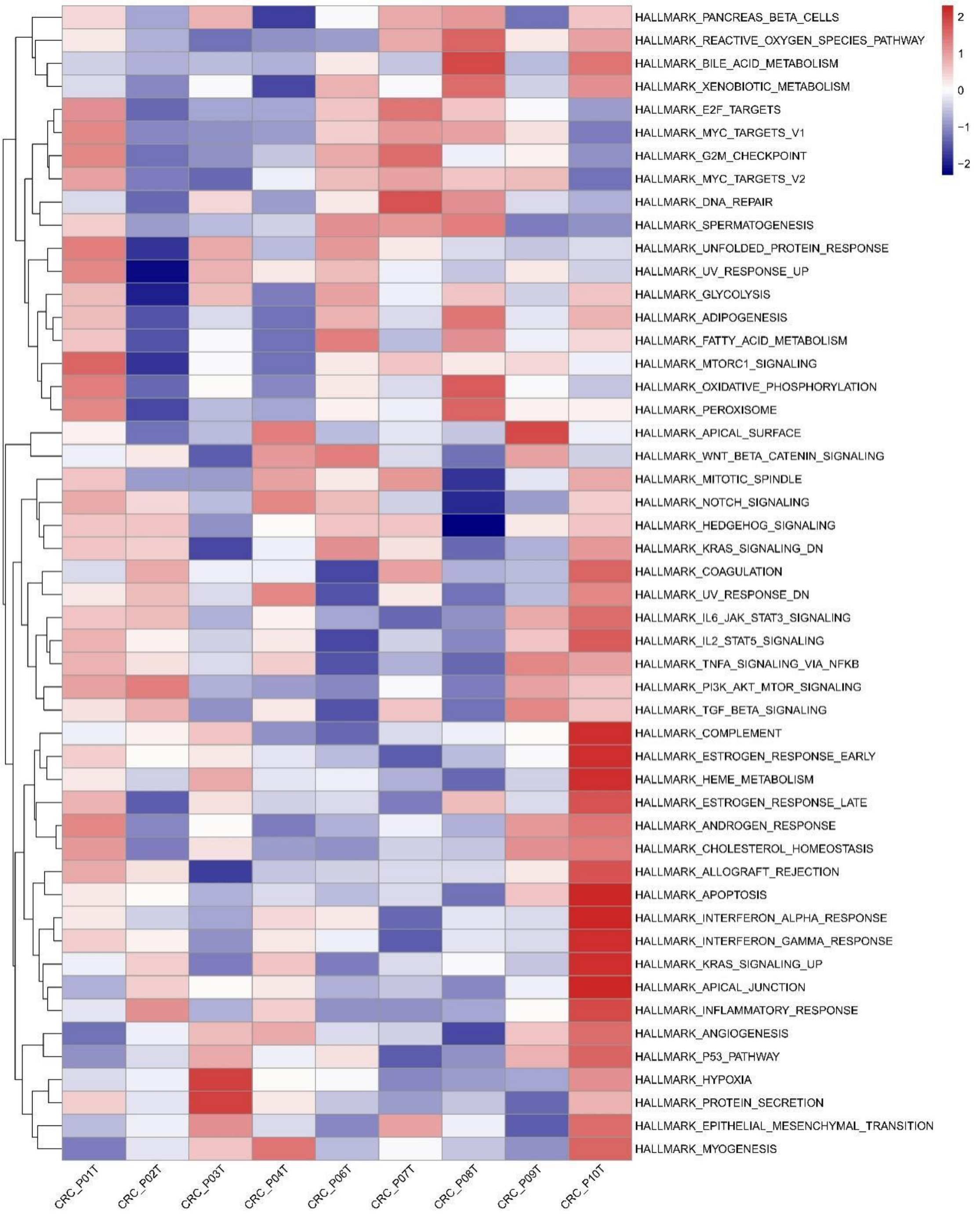

Figure S20. Heatmap of GSVA pathway activity in each tumour cell subtype from colorectal cancer (CRC). CRC_P09T with NFκB-active property (active TNFA_Signaling_via_NFκB) showed activity of gene set related to cell proliferation, such as G2M checkpoint, and higher activities of gene sets related to inflammation, such as IL2/6 signaling. CRC_P10T with Trail-active property showed activity of gene set related to apoptosis, such as Apoptosis signaling.

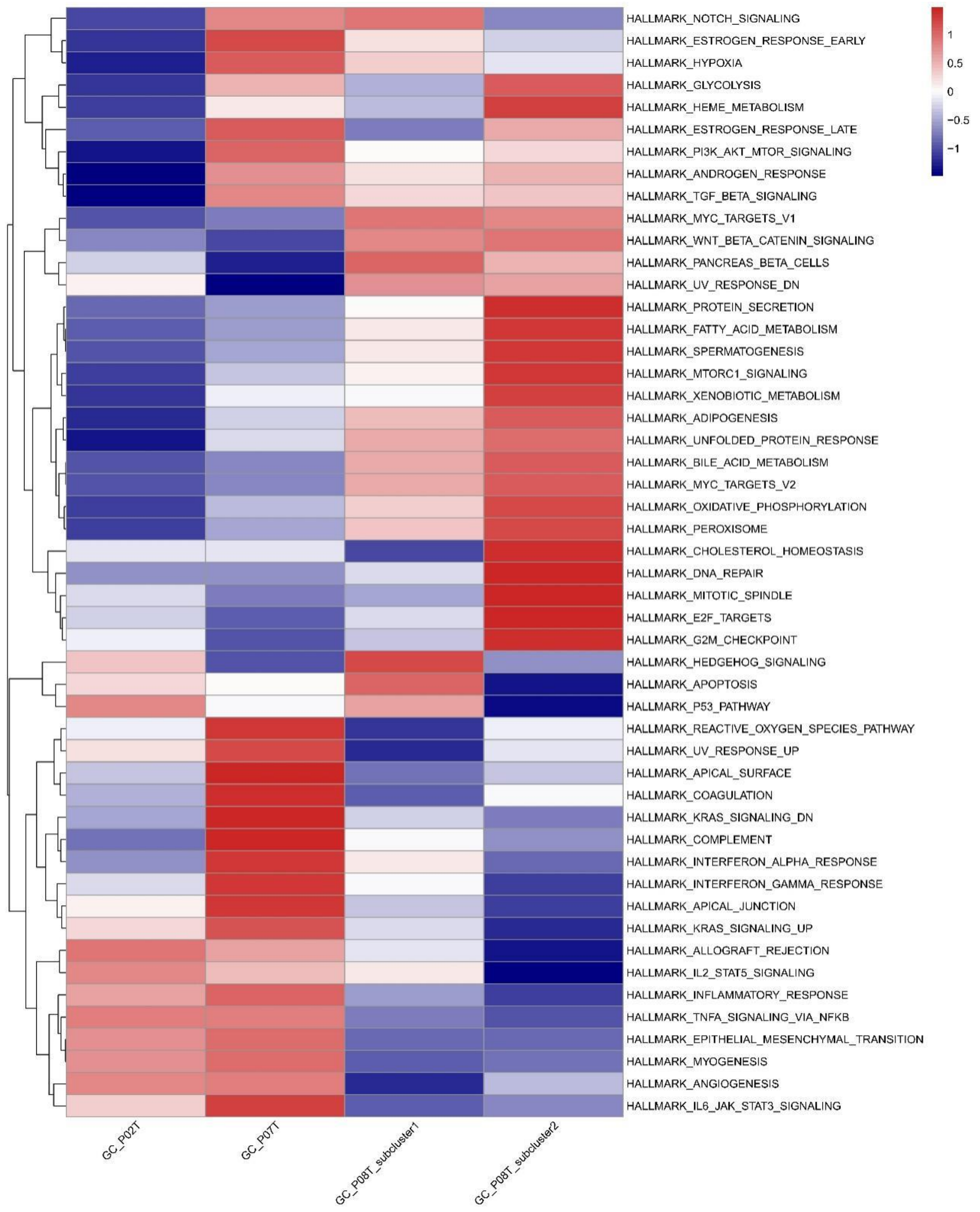

Figure S21. Heatmap of GSVA pathway activity in each tumour cell subtype from gastric cancer (GC). GC_P08T_1 with stem-like property showed activity (active WNT_Beta_Catenin_Signaling) of gene set related to the maintenance and survival of cancer stem cells (CSCs), such as Notch and Hedgehog signaling. GC_P02T and GC_P07T with NFκB-active property (active TNFA_Signaling_via_NFκB) showed higher activities of gene sets related to inflammation, such as inflammation response and IL2/6 signaling.

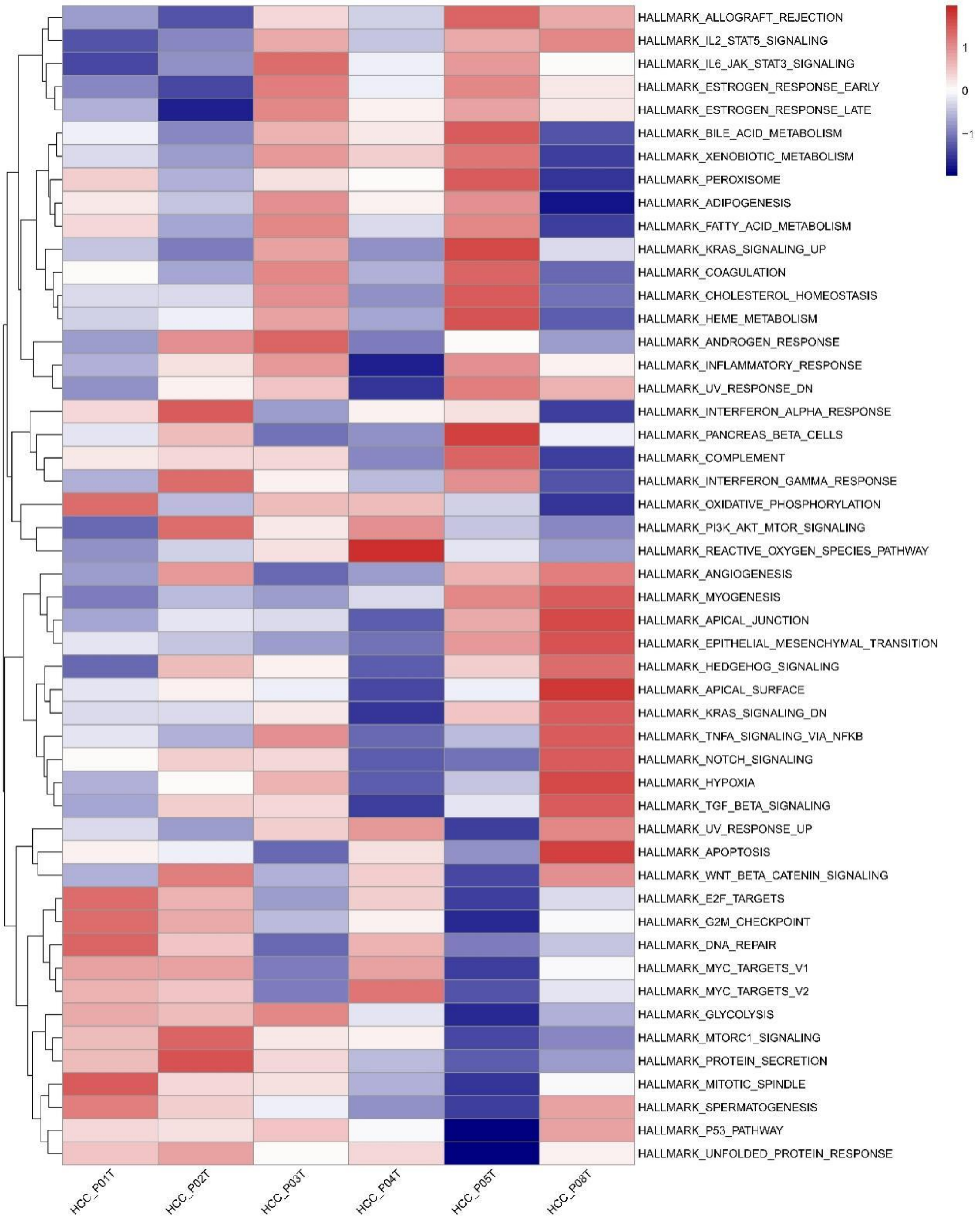

Figure S22. Heatmap of GSVA pathway activity in each tumour cell subtype from hepatocellular carcinoma (HCC). HCC_P03T and HCC_P08T with NFκB-active property (active TNFA_Signaling_via_NFκB) showed higher activities of gene sets related to inflammation, such as inflammation response and IL2/6 signaling.

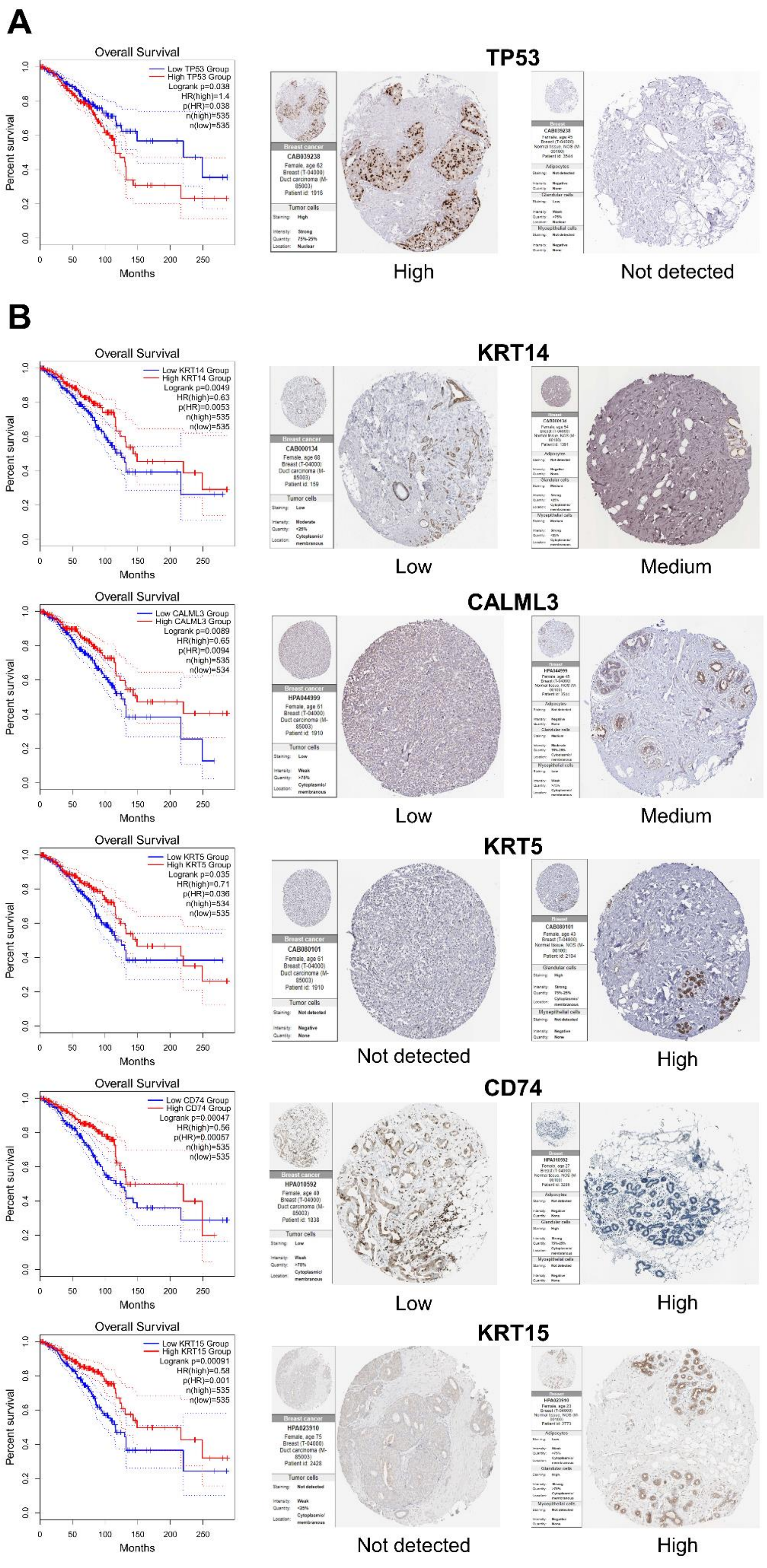
A
Overall Survival
Low TP53 Group
High TP53 Group
Logrank p=0.038
HR(high)=1.4
p(HR)=0.038
n(high)=535
n(low)=535
Percent survival
Months
TP53
High
Not detected
B
Overall Survival
Low KRT14 Group
High KRT14 Group
Logrank p=0.0049
HR(high)=0.63
p(HR)=0.0053
n(high)=535
n(low)=535
KRT14
Low
Medium
Overall Survival
Low CALML3 Group
High CALML3 Group
Logrank p=0.0089
HR(high)=0.65
p(HR)=0.0094
n(high)=535
n(low)=534
CALML3
Low
Medium
Overall Survival
Low KRT5 Group
High KRT5 Group
Logrank p=0.035
HR(high)=0.71
p(HR)=0.036
n(high)=534
n(low)=535
KRT5
Not detected
High
Overall Survival
Low CD74 Group
High CD74 Group
Logrank p=0.00047
HR(high)=0.56
p(HR)=0.00057
n(high)=535
n(low)=535
CD74
Low
High
Overall Survival
Low KRT15 Group
High KRT15 Group
Logrank p=0.00091
HR(high)=0.58
p(HR)=0.001
n(high)=535
n(low)=535
KRT15
Not detected
High

Figure S23. Specific biomarkers of tumour cell subtypes from breast cancer (BC). The biomarkers were classified into two groups based on the prognosis results of survival analysis. (A) Biomarkers with poorer prognosis. (B) Biomarkers with better prognosis. For each biomarker, the left image is the Kaplan-Meier curve plot, downloaded from GEPIA2 database. In survival analysis, a higher curve indicates this group has a higher survival rate, suggesting that high or low expression of this biomarker is beneficial for cancer patients' survival. Conversely, a lower curve signifies a lower survival rate. For each biomarker, the middle and right images were sourced from the HPA database, representing the protein expression of the biomarker in tumour tissue samples and normal tissue samples, respectively.

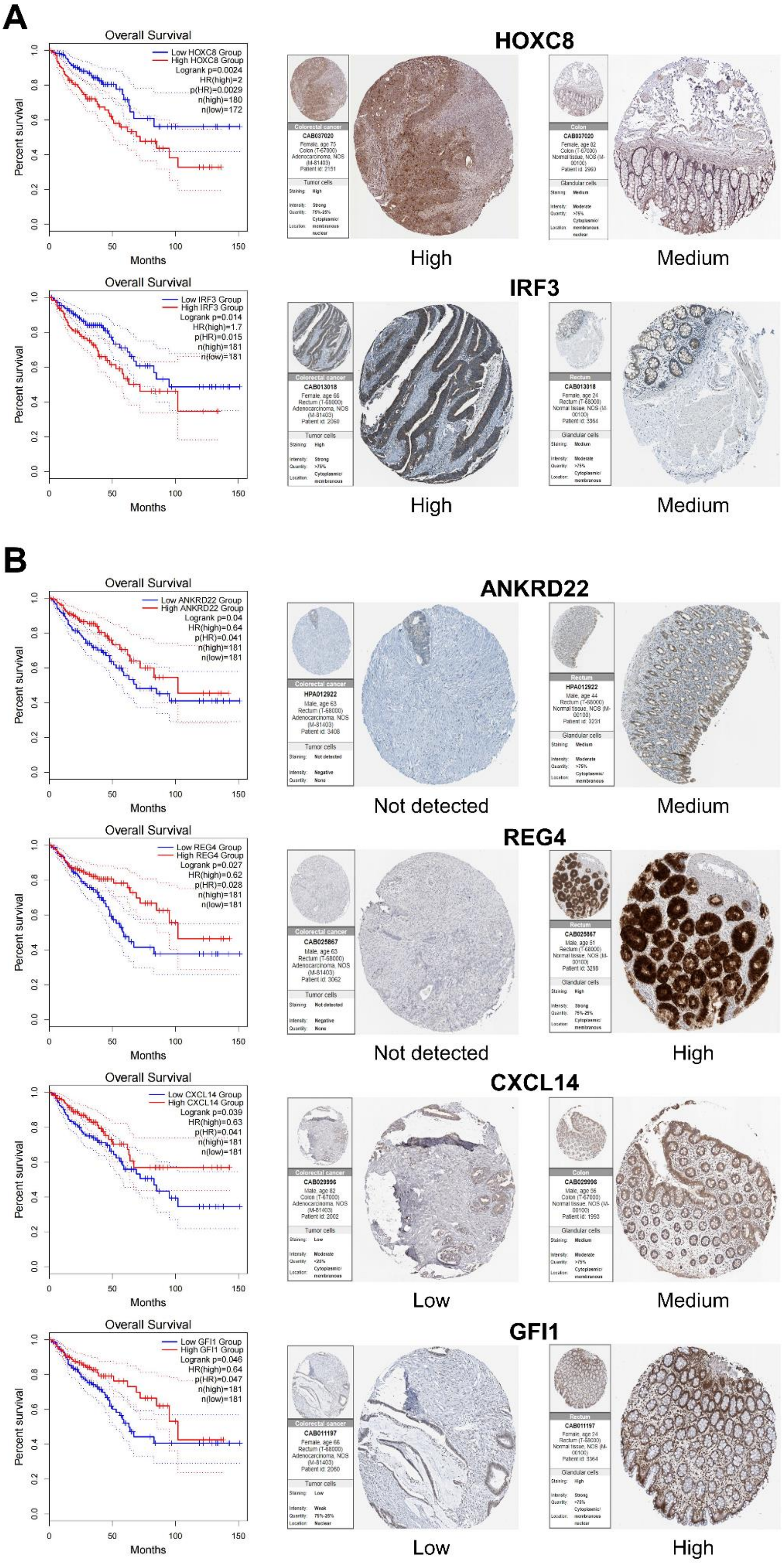
A
Overall Survival
Low HOXC8 Group
High HOXC8 Group
Logrank p=0.0024
HR(high)=2
p(HR)=0.0029
n(high)=180
n(low)=172
Percent survival
Months
HOXC8
High
Medium
Overall Survival
Low IRF3 Group
High IRF3 Group
Logrank p=0.014
HR(high)=1.7
p(HR)=0.015
n(high)=181
n(low)=181
Percent survival
Months
IRF3
High
Medium
B
Overall Survival
Low ANKRD22 Group
High ANKRD22 Group
Logrank p=0.04
HR(high)=0.64
p(HR)=0.041
n(high)=181
n(low)=181
Percent survival
Months
ANKRD22
Not detected
Medium
Overall Survival
Low REG4 Group
High REG4 Group
Logrank p=0.027
HR(high)=0.62
p(HR)=0.028
n(high)=181
n(low)=181
Percent survival
Months
REG4
Not detected
High
Overall Survival
Low CXCL14 Group
High CXCL14 Group
Logrank p=0.039
HR(high)=0.63
p(HR)=0.041
n(high)=181
n(low)=181
Percent survival
Months
CXCL14
Low
Medium
Overall Survival
Low GFI1 Group
High GFI1 Group
Logrank p=0.046
HR(high)=0.64
p(HR)=0.047
n(high)=181
n(low)=181
Percent survival
Months
GFI1
Low
High

Figure S24. Specific biomarkers of tumour cell subtypes from colorectal cancer (CRC). The biomarkers were classified into two groups based on the prognosis results of survival analysis. (A) Biomarkers with poorer prognosis. (B) Biomarkers with better prognosis. For each biomarker, the left image is the Kaplan-Meier curve plot, downloaded from GEPIA2 database. In survival analysis, a higher curve indicates this group has a higher survival rate, suggesting that high or low expression of this biomarker is beneficial for cancer patients' survival. Conversely, a lower curve signifies a lower survival rate. For each biomarker, the middle and right images were sourced from the HPA database, representing the protein expression of the biomarker in tumour tissue samples and normal tissue samples, respectively.

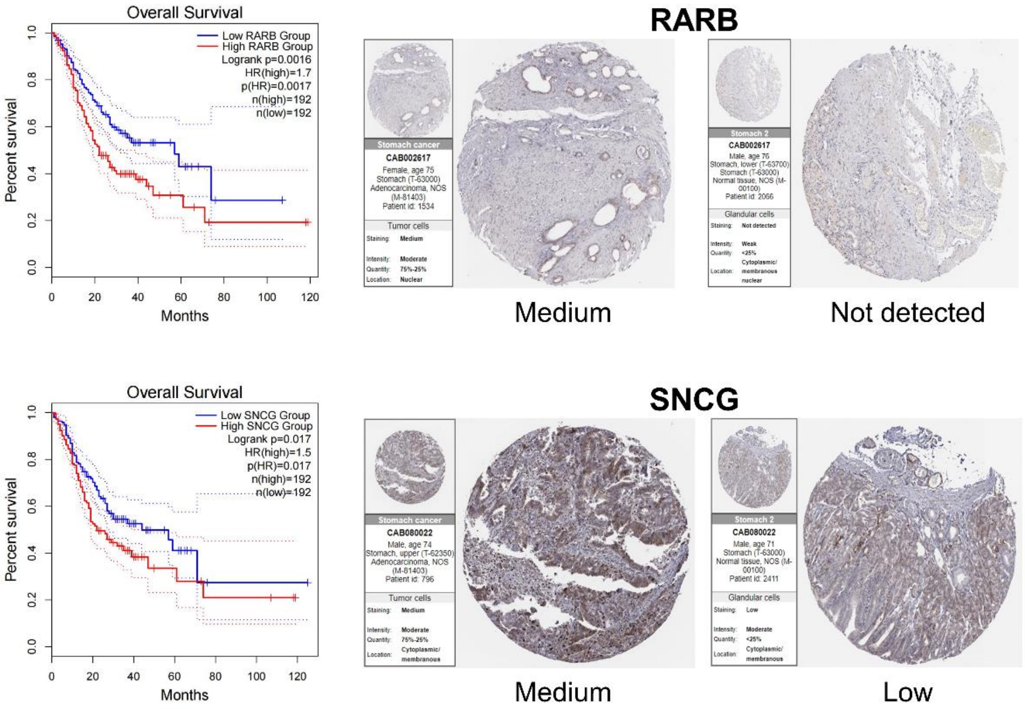

Figure S25. Specific biomarkers of tumour cell subtypes from gastric cancer (GC). The high expression of these biomarkers is indicative of a poorer prognosis. For each biomarker, the left image is the Kaplan-Meier curve plot, downloaded from GEPIA2 database. In survival analysis, a higher curve indicates this group has a higher survival rate, suggesting that high or low expression of this biomarker is beneficial for cancer patients' survival. Conversely, a lower curve signifies a lower survival rate. For each biomarker, the middle and right images were sourced from the HPA database, representing the protein expression of the biomarker in tumour tissue samples and normal tissue samples, respectively.

A

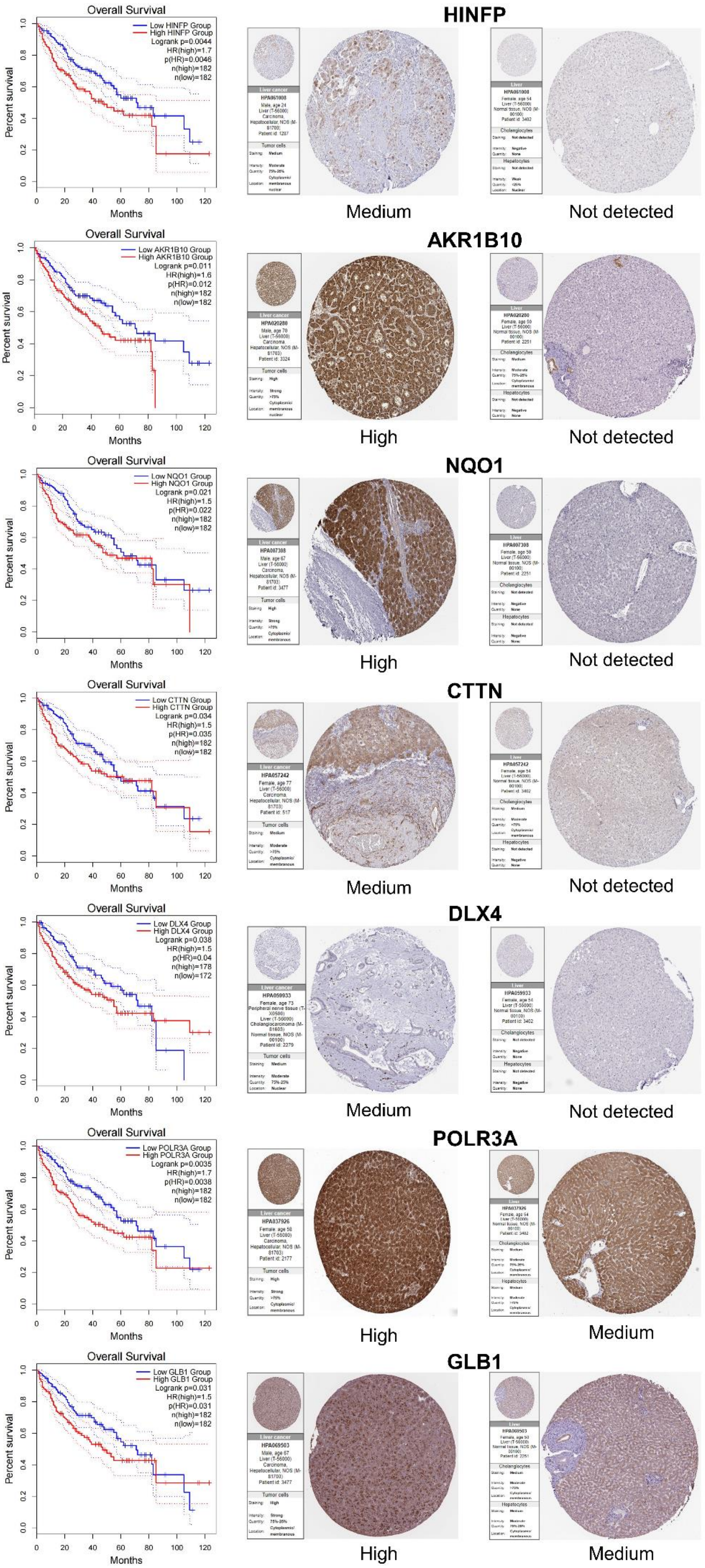
HINFP
Overall Survival
Low HINFP Group
High HINFP Group
Logrank p=0.0044
HR(high)=1.7
p(HR)=0.0046
n(high)=182
n(low)=182
Percent survival
Months
Medium
Not detected
AKR1B10
Overall Survival
Low AKR1B10 Group
High AKR1B10 Group
Logrank p=0.011
HR(high)=1.6
p(HR)=0.012
n(high)=182
n(low)=182
High
Not detected
NQO1
Overall Survival
Low NQO1 Group
High NQO1 Group
Logrank p=0.021
HR(high)=1.5
p(HR)=0.022
n(high)=182
n(low)=182
High
Not detected
CTTN
Overall Survival
Low CTTN Group
High CTTN Group
Logrank p=0.034
HR(high)=1.5
p(HR)=0.035
n(high)=182
n(low)=182
Medium
Not detected
DLX4
Overall Survival
Low DLX4 Group
High DLX4 Group
Logrank p=0.038
HR(high)=1.5
p(HR)=0.04
n(high)=178
n(low)=172
Medium
Not detected
POLR3A
Overall Survival
Low POLR3A Group
High POLR3A Group
Logrank p=0.0035
HR(high)=1.7
p(HR)=0.0038
n(high)=182
n(low)=182
High
Medium
GLB1
Overall Survival
Low GLB1 Group
High GLB1 Group
Logrank p=0.031
HR(high)=1.5
p(HR)=0.031
n(high)=182
n(low)=182
High
Medium

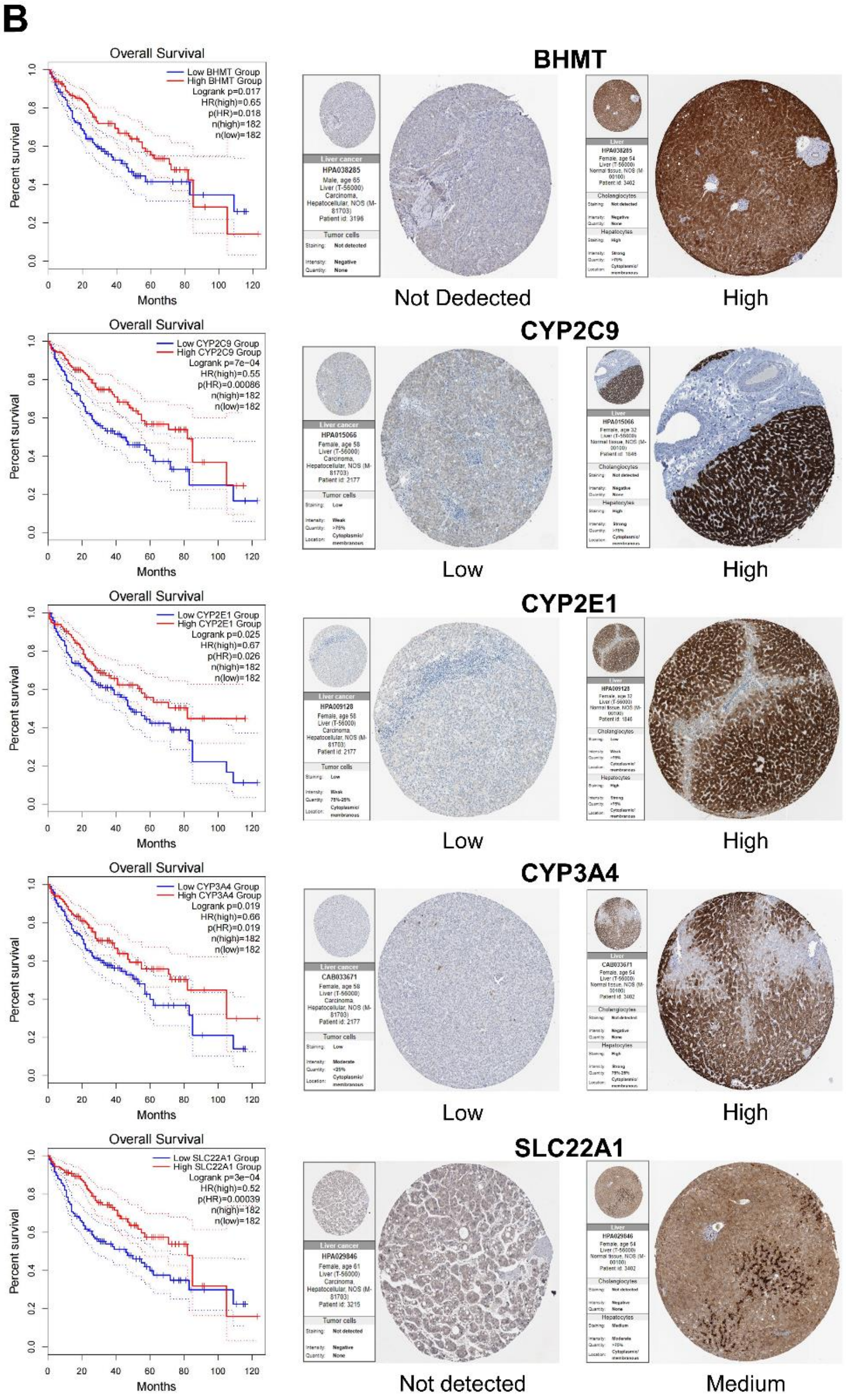

Figure S26. Specific biomarkers of tumour cell subtypes from hepatocellular carcinoma (HCC). The biomarkers were classified into two groups based on the prognosis results of survival analysis. (A) Biomarkers with poorer prognosis. (B) Biomarkers with better prognosis. For each biomarker, the left image is the Kaplan-Meier curve plot, downloaded from GEPIA2 database. In survival analysis, a higher curve indicates this group has a higher survival rate, suggesting that high or low expression of this biomarker is beneficial for cancer patients' survival. Conversely, a lower curve signifies

a lower survival rate. For each biomarker, the middle and right images were sourced from the HPA database, representing the protein expression of the biomarker in tumour tissue samples and normal tissue samples, respectively.

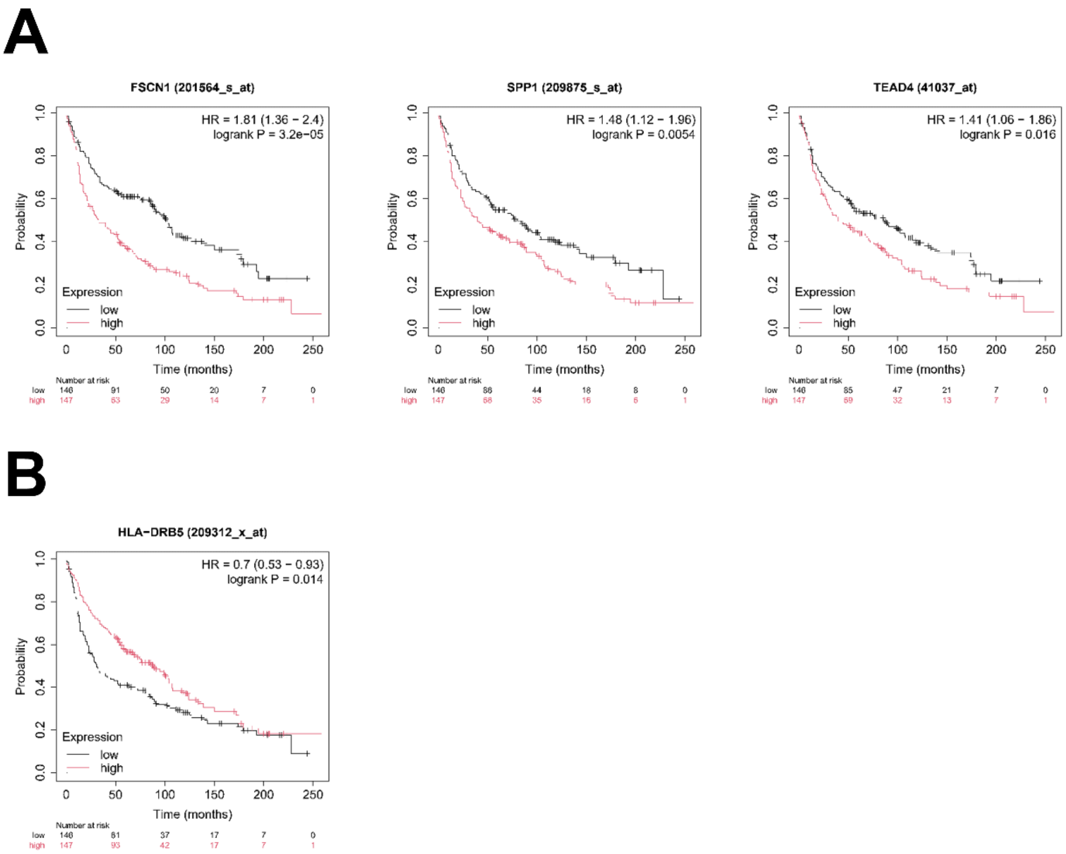

Figure S27. Specific biomarkers of tumour cell subtypes from lung adenocarcinoma (LUAD) after validation of independent datasets. The biomarkers were classified into two groups based on the prognosis results of survival analysis. (A) Biomarkers with poorer prognosis. (B) Biomarkers with better prognosis. For each biomarker, the Kaplan-Meier curve plot was downloaded from the Kaplan-Meier Plotter database, excluding The Cancer Genome Atlas (TCGA) LUAD datasets. In survival analysis, a higher curve indicates this group has a higher survival rate, suggesting that high or low expression of this biomarker is beneficial for cancer patients' survival. Conversely, a lower curve signifies a lower survival rate.

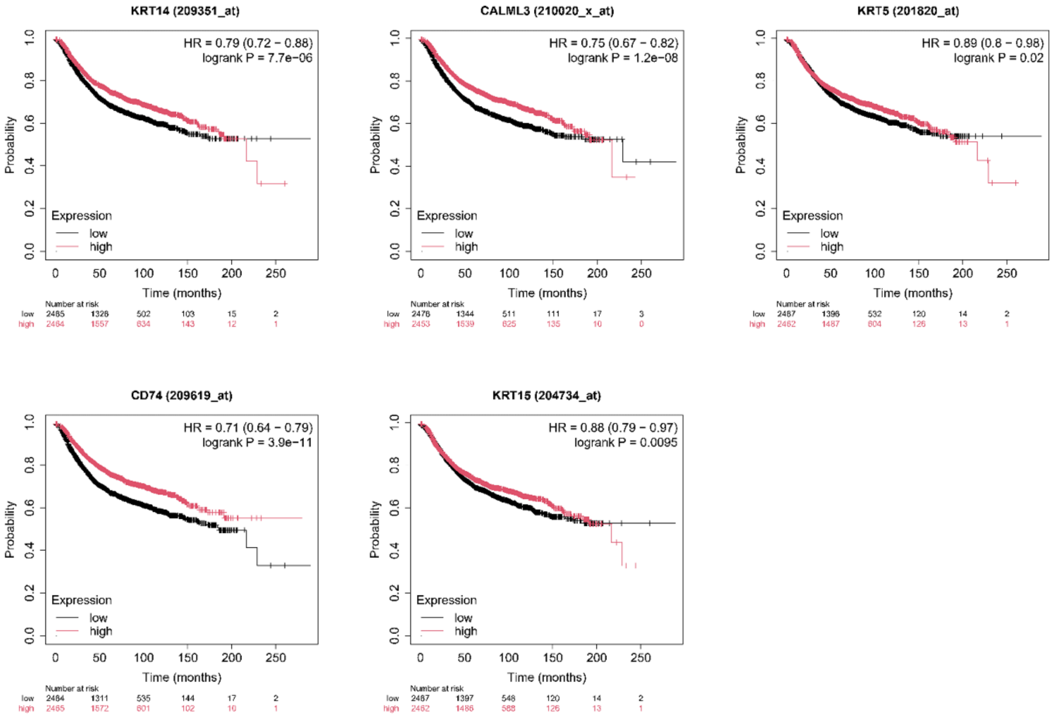

Figure S28. Specific biomarkers of tumour cell subtypes from breast cancer (BC) after validation of independent datasets. The high expression of these biomarkers is indicative of a better prognosis. For each biomarker, the Kaplan-Meier curve plot was downloaded from the Kaplan-Meier Plotter database, excluding The Cancer Genome Atlas (TCGA) BC datasets. In survival analysis, a higher curve indicates this group has a higher survival rate, suggesting that high or low expression of this biomarker is beneficial for cancer patients' survival. Conversely, a lower curve signifies a lower survival rate.

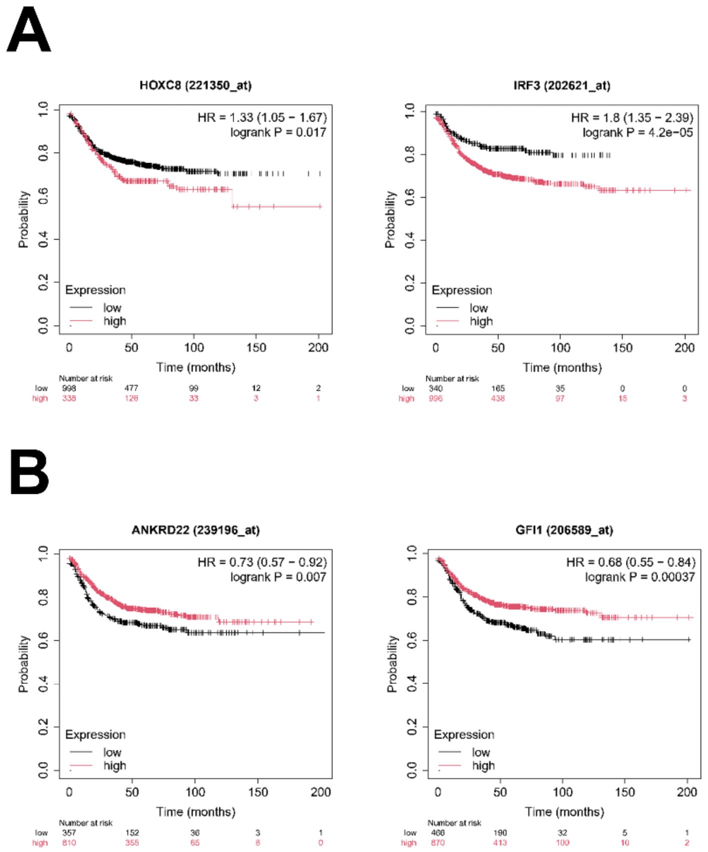

Figure S29. Specific biomarkers of tumour cell subtypes from colorectal cancer (CRC) after validation of independent datasets. (A) Biomarkers with poorer prognosis. (B) Biomarkers with better prognosis. For each biomarker, the Kaplan-Meier curve plot was downloaded from the Kaplan-Meier Plotter database, excluding The Cancer Genome Atlas (TCGA) CRC datasets. In survival analysis, a higher curve indicates this group has a higher survival rate, suggesting that high or low expression of this biomarker is beneficial for cancer patients' survival. Conversely, a lower curve signifies a lower survival rate.

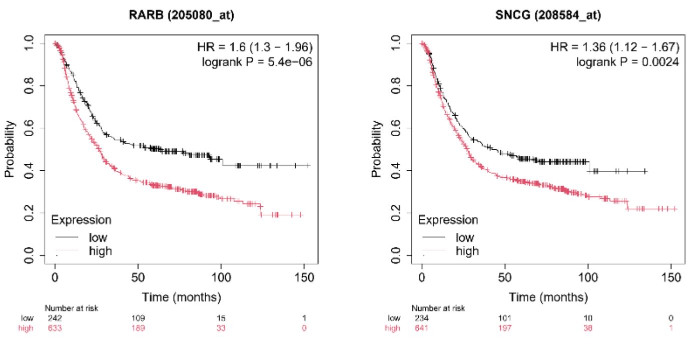

Figure S30. Specific biomarkers of tumour cell subtypes from gastric cancer (GC) after validation of independent datasets. The high expression of these biomarkers is indicative of a poorer prognosis. For each biomarker, the Kaplan-Meier curve plot was downloaded from the Kaplan-Meier Plotter database, excluding The Cancer Genome Atlas (TCGA) GC datasets. In survival analysis, a higher curve indicates this group has a higher survival rate, suggesting that high or low expression of this biomarker is beneficial for cancer patients' survival. Conversely, a lower curve signifies a lower survival rate.

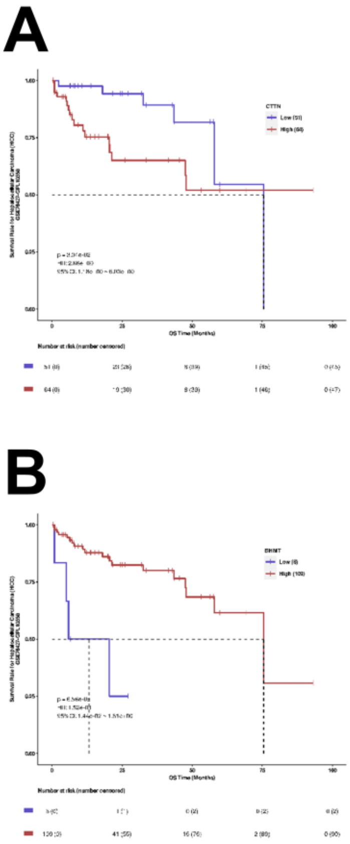

Figure S31. Specific biomarkers of tumour cell subtypes from hepatocellular carcinoma (HCC) after validation of independent datasets. (A) Biomarkers with poorer prognosis. (B) Biomarkers with better prognosis. For each biomarker, the Kaplan-Meier curve plot was downloaded from the PanCanSurvPlot database, excluding The Cancer Genome Atlas (TCGA) HCC datasets. In survival analysis, a higher curve indicates this group has a higher survival rate, suggesting that high or low expression of this biomarker is beneficial for cancer patients' survival. Conversely, a lower curve signifies a lower survival rate.
